# Identification and characterization of a strong constitutive promoter *stnYp* for activating biosynthetic genes and producing natural products in *streptomyces*

**DOI:** 10.1186/s12934-023-02136-9

**Published:** 2023-07-13

**Authors:** Wenli Guo, Zhihong Xiao, Tingting Huang, Kai Zhang, Hai-Xue Pan, Gong-Li Tang, Zixin Deng, Rubing Liang, Shuangjun Lin

**Affiliations:** 1 State Key Laboratory of Microbial Metabolism, Joint International Research Laboratory on Metabolic & Developmental Sciences, School of Life Sciences & Biotechnology, Shanghai Jiao Tong University, 800 Dongchuan Road, Shanghai, 200240 China; 2 Haihe Laboratory of Synthetic Biology, Tianjin, 300308 China; 3 State Key Laboratory of Bioorganic and Natural Products Chemistry, Center for Excellence in Molecular Synthesis, Shanghai Institute of Organic Chemistry, University of Chinese Academy of Sciences, Chinese Academy of Sciences, Shanghai, 200032 China; 4 Frontiers Science Center for Transformative Molecules, Shanghai Jiao Tong University, 800 Dongchuan Road, Shanghai, 200240 China

**Keywords:** Natural products, *Streptomyces*, Constitutive promoter, *stnYp*, Heterologous expression, Biosynthetic gene cluster

## Abstract

**Background:**

*Streptomyces* are well known for their potential to produce various pharmaceutically active compounds, the commercial development of which is often limited by the low productivity and purity of the desired compounds expressed by natural producers. Well-characterized promoters are crucial for driving the expression of target genes and improving the production of metabolites of interest.

**Results:**

A strong constitutive promoter, *stnYp*, was identified in *Streptomyces flocculus* CGMCC4.1223 and was characterized by its effective activation of silent biosynthetic genes and high efficiency of heterologous gene expression. The promoter *stnYp* showed the highest activity in model strains of four *Streptomyces* species compared with the three frequently used constitutive promoters ermEp*, kasOp*, and *SP44*. The promoter *stnYp* could efficiently activate the indigoidine biosynthetic gene cluster in *S. albus* J1074, which is thought to be silent under routine laboratory conditions. Moreover, *stnYp* was found suitable for heterologous gene expression in different *Streptomyces* hosts. Compared with the promoters ermEp*, kasOp*, and *SP44*, *stnYp* conferred the highest production level of diverse metabolites in various heterologous hosts, including the agricultural-bactericide aureonuclemycin and the antitumor compound YM-216391, with an approximately 1.4 − 11.6-fold enhancement of the yields. Furthermore, the purity of tylosin A was greatly improved by overexpressing rate-limiting genes through *stnYp* in the industrial strain. Further, the yield of tylosin A was significantly elevated to 10.30 ± 0.12 g/L, approximately 1.7-fold higher than that of the original strain.

**Conclusions:**

The promoter *stnYp* is a reliable, well-defined promoter with strong activity and broad suitability. The findings of this study can expand promoter diversity, facilitate genetic manipulation, and promote metabolic engineering in multiple *Streptomyces* species.

**Supplementary Information:**

The online version contains supplementary material available at 10.1186/s12934-023-02136-9.

## Background

Natural products are major sources of drug molecules, including antibacterial, antiviral, antifungal, antiparasitic, antitumor, and immunosuppressive compounds [1, 2]. *Streptomyces*, gram-positive filamentous bacteria with extraordinarily high GC-content genomes and intricate regulatory networks, are notable for their central role in discovering and producing natural products [3, 4]. Genes responsible for the biosynthesis of natural products are generally clustered in a continuous genome region, termed biosynthetic gene clusters (BGCs) [5]. Many microbial genome sequencing studies have shown that there are generally 25–50 BGCs in a single *Streptomyces* genome, whereas most of them are silent or cryptic under conventional culture conditions [5, 6]. Secondary metabolites in native producers are usually produced at a low titer owing to restricted transcription and tight regulation in response to environmental conditions [7]. Regarding scale-up production, the yield of natural products from native producers is far from meeting industrial needs [8]. Therefore, unlocking silent genes or regulating target genes to activate or enhance the production of natural products has become a popular research topic in this field. Moreover, because many of the original microorganisms producing bioactive natural products are usually slow growing and difficult to operate, it is desirable to transfer the biosynthetic pathway of interest into a well-established heterologous host for genetic manipulation and robust overproduction [9]. Heterologous expression has been widely used to activate and manipulate silent BGCs to efficiently produce desired metabolites or improve their pharmacological properties [10, 11]. Several *Streptomyces* strains have been used as surrogate hosts for BGC expression, such as *S. coelicolor*, *S. lividans*, *S. albus*, and *S. venezuelae* [12, 13]. However, the regulatory elements and metabolites produced by different heterologous hosts are diverse.

Promoters are key elements of both genetic manipulation and metabolic engineering [5]. Replacing native promoters with universal constitutive promoters or host-specific strong promoters is an effective strategy for activating silent natural product biosynthetic pathways in native producers or improving the titer of target natural products in heterologous hosts [8, 9]. However, the limited number of reliable constitutive promoters is a major drawback for the efficient initiation and heterologous expression of target genes in *Streptomyces* [14]. Promoter ermEp* from *Saccharopolyspora erythraea* is the most widely used constitutive promoter, generated from the promoter of the *ermE* gene with a trinucleotide deletion [15, 16]. Promoter *SF14p* was isolated from the phage I19 genome of *S. ghanaensis* [17]. Another engineered promoter, kasOp*, exhibits higher activity than ermEp* and *SF14p*, derived from the promoter of *kasO* (*sco6280*) encoding a regulator of *Streptomyces* antibiotic regulatory protein family in *S. coelicolor* [18]. In addition, a series of synthetic promoter libraries have been constructed by mimicking certain promoters, such as *emrEp1* and kasOp* [19, 20]. Of these promoters, *SP44* is the strongest constitutive promoter engineered from the promoter kasOp*, with twice the activity of the latter [14]. Therefore, it is imperative to explore and develop diverse, effective, and well-characterized promoters suitable for heterologous gene expression and natural product synthesis in *Streptomyces*.

This study identified and characterized a strong constitutive promoter, *stnYp*, in the antitumor agent streptonigrin-producing *S. flocculus* CGMCC4.1223. Its activity was determined using the gene *xylE* as a reporter, and its strength was compared with that of three widely used promoters (ermEp*, kasOp*, and *SP44*). The suitability of *stnYp* for heterologous gene expression was tested in strains of four *Streptomyces* species (*S. albus* J1074, *S. coelicolor* M1154, *S. lividans* TK24, and *S. venezuelae* ISP5230). Furthermore, its efficiency in activating the silent gene cluster (indigoidine gene cluster) and the expression of multicistronic biosynthetic cassettes (aureonuclemycin and YM-216391 gene clusters) were determined. Its application in eliminating tylosin A by-products from the industrial strain was also characterized. These findings replenish the promoter pool for the effective expression of biosynthetic genes and benefit the development and commercialization of valuable natural products in *Streptomyces* cell factories.

## Results

### The promoter ***stnYp*** is a strong, constitutive promoter with a conserved structure

**Fig. 1:**
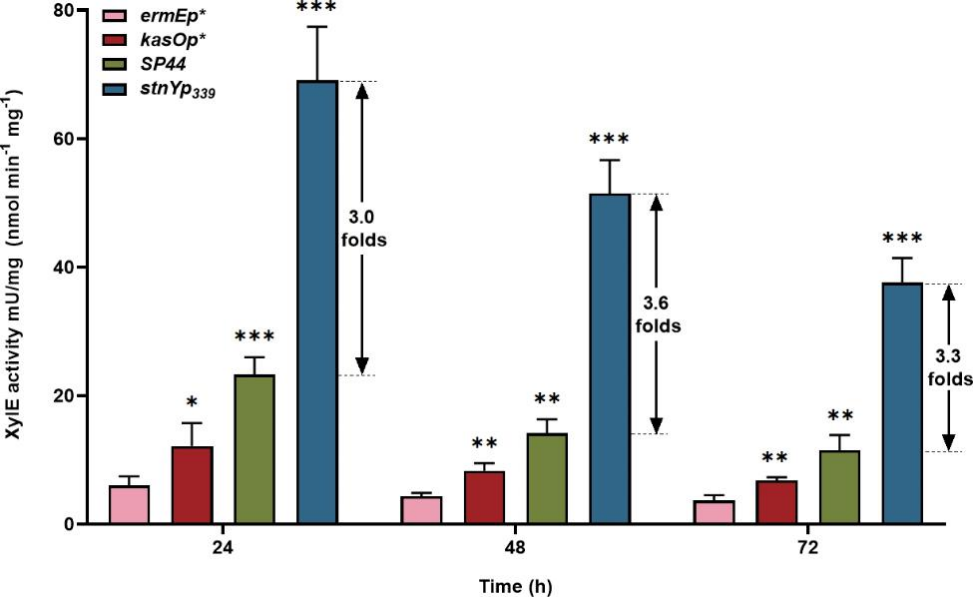
Activity evaluation of promoter *stnYp*_*339*_ in *S. albus* J1074. The activity of promoter *stnYp*_*339*_ was evaluated with the XylE assay. XylE assay was performed using samples harvested after 24 h, 48 and 72 h culture. The activities of three promoters (ermEp*, kasOp* and *SP44*) were also detected and used for comparison. Statistical analyses were performed and marked (*, *p* ≤ 0.1; **, *p* ≤ 0.05; ***, *p* ≤ 0.01)

Bacterial promoters essential for transcription initiation are short DNA sequences with unclear or ambiguous boundaries [21]. Native promoters are usually located in an intergenic DNA region near the transcription start site (TSS), upstream of the 5’ end of the functional gene [22]. StnY is a member of the CopG family of transcriptional regulators in *Streptomyces* and probably plays an important regulatory role in streptonigrin biosynthesis [23]. The upstream region of the putative *stnY* promoter spans more than 300 bp of DNA. The ability of the putative *stnY*promoter (designated *stnYp*_*339*_) to drive gene expression was determined using the gene *xylE* as a reporter. The gene *xylE* encodes catechol 2,3-dioxygenase, catalyzing the conversion of colorless catechol into yellow 2-hydroxymuconic semialdehyde, normally used as an effective reporter gene in different *Streptomyces* [24]. The proposed promoter region of the *stnY* promoter (*stnYp*_*339*_) and three other well-characterized constitutive promoters (ermEp*, kasOp*, and *SP44*) were assembled into reporter plasmid pDR3, generating plasmids pDR3-*stnYp*_*339*_, pDR3-ermEp*, pDR3-kasOp*, and pDR3-*SP44*, respectively. The resulting constructs were integrated into the *S. albus* J1074 strain, a well-characterized heterologous host with a rapid growth rate and easy genetic manipulation [25]. Since the same RBS sequence was used for all four promoters, the amount of XylE activity correlated with promoter strength. The results showed that the XylE activity in the control of the promoter *stnYp*_*339*_ was significantly higher than those of the other three promoters, both in the exponential (24 h) and stationary phases (48 and 72 h) (Fig. 1). The transcriptional level of the gene *xylE* under the control of the promoter *stnYp*_*339*_ was also significantly higher than those of the other three promoters in all growth phases (Additional file 1: Fig. S1). This demonstrates that the promoter *stnYp*_*339*_ was significantly stronger than ermEp*, kasOp*, and *SP44* in *S. albus* J1074.

The promoter sequence plays a critical role in promoter activity [26]. The structure of the promoter *stnYp*_*339*_ was analyzed based on the TSS and translation start sites (TLSs) in the genome sequence of *S. coelicolor* A3 and *S. lividans* TK24 [27, 28]. The results revealed conserved − 10 (TAnnnT) and weakly conserved − 35 motifs (nTGACn) in the promoter region of the *stnY* gene. The components of the promoter *stnYp*_*339*_ were also deduced from the promoter prediction database SAPPHIRE [29]. The core sequence of the promoter *stnYp*_*339*_ in the − 10 motifs is TAGCAT with extended − 10 motifs (TGC), similar to the consensus sequence. The core sequence of the promoter *stnYp*_*339*_ in the − 35 motifs is TTGGCG, replacing A with G in the fourth position relative to the conserved sequence. Spaces between − 10 and − 35 motifs are 17 nt, a typical length for *Streptomyces* promoters (Additional file 1: Fig. S2). Further, the TSS site of the promoter *stnYp*_*339*_ was determined by rapid amplification of 5’-cDNA ends (5’ RACE). The results showed that the TSS site of the *stnY* gene is in position − 16 concerning the initiation codon (Additional file 1: Fig. S2). In short, the sequence of the promoter *stnYp*_*339*_ was consistent with the consensus sequences, likely to be recognized by the essential housekeeping sigma (σ) factor HrdB in *Streptomyces* [30].

### The minimal promoter ***stnYp*** exhibits high activity for XylE expression

**Fig. 2:**
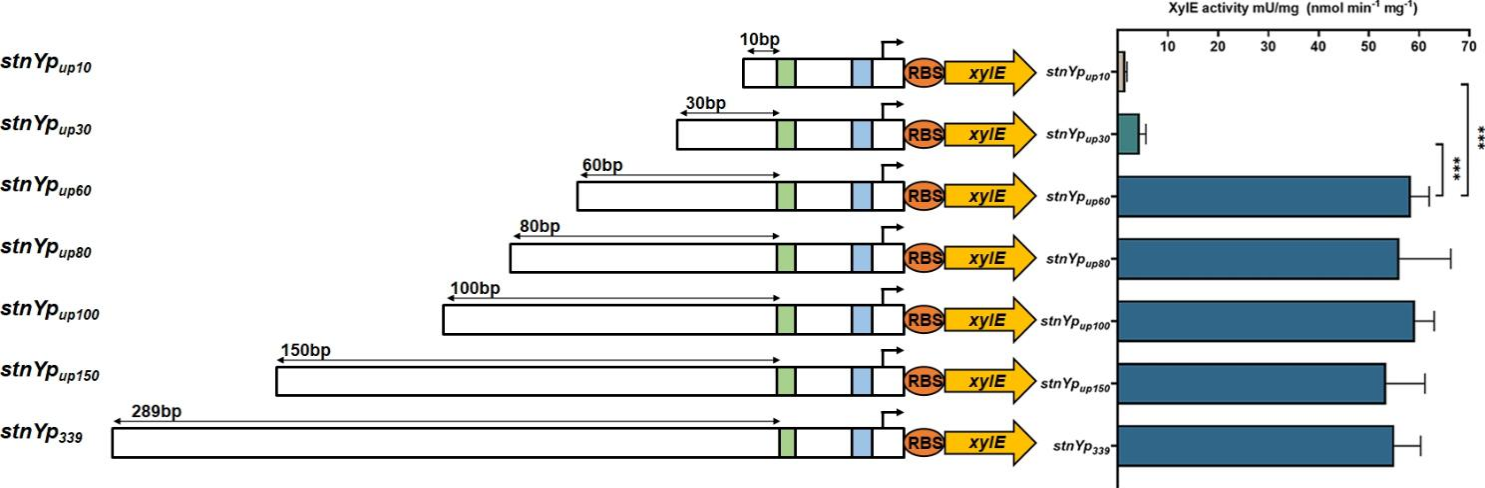
Determination of the minimal *stnYp* promoter. The schematic diagram of the reporter constructs harboring the full-length promoter *stnYp*_*339*_ and truncated mutants. Here, the marked 289 bp, 150 bp, 100 bp, 80 bp, 60 bp, 30 bp and 10 bp represented the upstream DNA length of the − 35 region (TTGGCG), corresponding to *stnYp*_*339*_, *stnYpup*_*150*_, *stnYpup*_*100*_, *stnYpup*_*80*_, *stnYpup*_*60*_, *stnYpup*_*30*_ and *stnYpup*_*10*_, respectively. The activities of truncated promoters were evaluated using *xylE* as a reporter gene in *S. albus* J1074. XylE assay was carried out with samples harvested after 24 h culture. Statistical analyses were performed and marked (***, *p* ≤ 0.01)

The upstream regions of the − 35 motifs interacting with the α-subunit carboxyl-terminal domain (αCTD) of RNA polymerase (RNAP) may contain additional specific DNA targets recognized by transcription factors detrimental to the application of gene circuits [31]. It is highly desirable to delineate the minimal promoter sequences and exclude unessential DNA regions potentially affecting gene expression. To identify the essential region and minimize the length of the promoter *stnYp*_*339*_, six 5’-truncated promoters of *stnYp*_*339*_ (designated as *stnYp*_*up150*_, *stnYp*_*up100*_, *stnYp*_*up80*_, *stnYp*_*up60*_, *stnYp*_*up30*,_ and *stnYp*_*up10*_) were constructed, and their activities were compared using the XylE assay in *S. albus* J1074 after 24 h culture. The results indicated that XylE activities under truncated promoters of *stnYp*_*up150*_, *stnYp*_*up100*_, *stnYp*_*up80*_, and *stnYp*_*up60*_ were not significantly different from those of the full-length promoter *stnYp*_*339*_. However, the activities of the promoters *stnYp*_*up30*_ and *stnYp*_*up10*_ dropped dramatically, with only 8.0% and 2.6% activity, respectively, compared with the promoter *stnYp*_*339*_ (Fig. 2). These data suggest that the upstream 60 bp motifs in the promoter *stnYp*_*339*_ are essential for its activity. Thus, the minimal length of the promoter *stnYp*_*339*_ was narrowed to 110 bp, ranging from upstream 60 bp of the − 35 motifs to downstream 21 bp of the − 10 motifs, and the promoter *stnYp*_*up60*_ was designated as *stnYp*.

### The promoter ***stnYp*** achieves efficient gene expression in various ***Streptomyces*** species

**Fig. 3:**
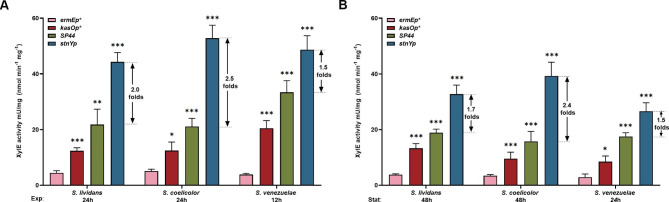
Activity evaluation of promoter *stnYp* in different *Streptomyces* hosts. Activities of promoters ermEp*, kasOp*, SP44 and *stnYp* in *S. lividans* TK24, *S. coelicolor* M1154 and *S. venezuelae* ISP5230 were evaluated with the XylE assay. **(A)** All cultures were harvested and sampled at the exponential phase (24 h from *S. lividans* and *S. coelicolor* and 12 h from *S. venezuelae*) to detect XylE activity. **(B)** All cultures were harvested and sampled at the stationary phase (48 h from *S. lividans* and *S. coelicolor* and 24 h from *S. venezuelae*) to detect XylE activity. Statistical analyses were performed and marked (*, *p* ≤ 0.1; **, *p* ≤ 0.05; ***, *p* ≤ 0.01)

The suitability of promoters in different hosts is critical for their use in genetic manipulation and industrial applications. The strength of the promoter *stnYp* in strains of three *Streptomyces* model species (*S. coelicolor* M1154, *S. lividans* TK124, and *S. venezuelae* ISP5230) was evaluated and compared with that of the three promoters (ermEp*, kasOp*, and *SP44*) using the XylE assay. The experimental designs for the three species were the same as those for *S. albus*. Samples were harvested in exponential and stationary growth phases: after the 24 and 48 h culture of *S. lividans* TK24 and *S. coelicolor* M1154, and after the 12 and 24 h culture of *S. venezuelae* ISP5230. Similar to the results obtained from *S. albus* J1074, the promoter *stnYp* conferred higher XylE activity than the promoters ermEp*, kasOp*, and *SP44* in both exponential and stationary growth phases in all three strains, particularly in *S. coelicolor* M1154 (Fig. 3A and B). These results indicate that the promoter *stnYp* is suitable for the stable and efficient expression of heterologous genes in various *Streptomyces* hosts and that the activity of the promoter *stnYp* is unaffected by intracellular differences.

### Engineering the silent BGC of indigoidine with promoter ***stnYp*** and effectively enhancing indigoidine production in ***S. albus***

**Fig. 4:**
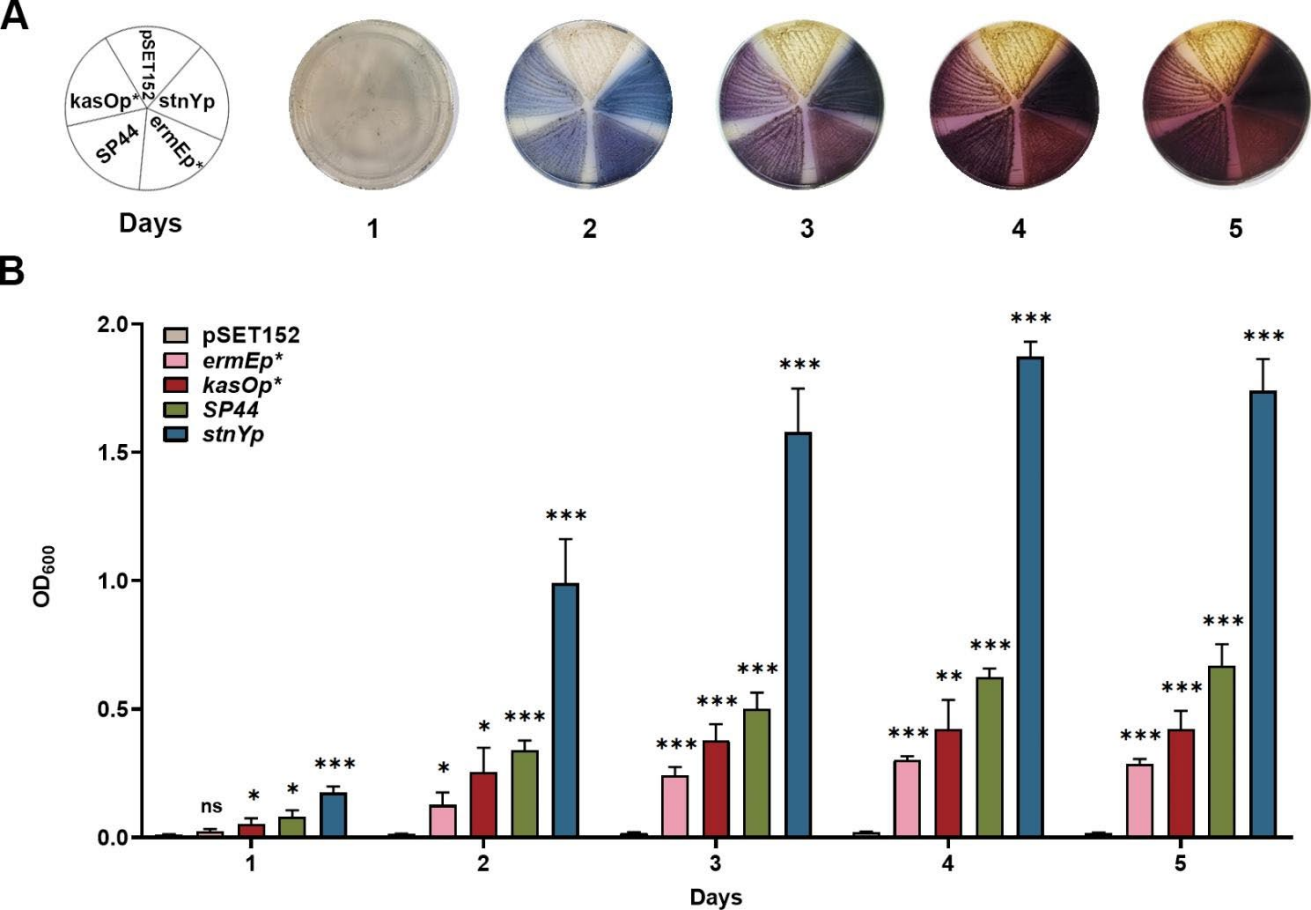
Application of promoter *stnYp* for indigoidine production. **(A)** Visual observation of indigoidine production on R5A agar medium from recombinant strain harboring *indC* gene under control of *stnYp* promoter and other promoters (ermEp*, kasOp* and *SP44*). The empty plasmid pSET152 was introduced into *S. albus* J1074 as a negative control. The plates were photographed every day in 5-day cultivation (1–5 days). Representative images of three independent experiments with similar results were shown. **(B)** Indigoidine production in R5A liquid media from recombinant strains (*S. albus*-ermEp*-*indC*, *S. albus*-kasOp*-*indC*, *S. albus*-*SP44*-*indC*, *S. albus*-*stnYp*-*indC*) harboring the *indC* gene under the control of ermEp*, kasOp*, *SP44* and *stnYp* were monitored, and empty plasmid pSET152 was introduced into *S. albus* J1074 as a negative control. Indigoidine production was measured by detecting OD_600_ of the 10-fold diluted supernatant of fermentation cultures with DMSO. Fermentation was carried out for five days and the cultures were collected every day. Three independent experiments were carried out for each strain and all data were the average data. Statistical analyses were performed and marked (*, *p* ≤ 0.1; **, *p* ≤ 0.05; ***, *p* ≤ 0.01)

The BGC of indigoidine is considered silent in *S. albus* J1074, acting as a representative of silent gene clusters to be activated [32]. The activation of silent BGC by the promoter *stnYp* was analyzed by comparing its stimulation efficiency for indigoidine synthesis with that of other promoters. The *indC* gene was cloned under the control of the promoter *stnYp* in plasmid pSET152 and then introduced into *S. albus* J1074 for heterologous expression. The three constitutive promoters, ermEp*, kasOp*, and *SP44*, were used as references, and the empty plasmid pSET152 was used as a negative control. After 5 days of cultivation in the R5A medium, indigoidine production of different derivatives was monitored every 24 h. The results showed that *S. albus* J1074 containing a plasmid with *indC* under the promoter *stnYp* (*S. albus*-*stnYp*-*indC*) exhibited a much darker blue color than the other three strains (*S. albus*-ermEp*-*indC*, *S. albus*-kasOp*-*indC*, *S. albus*-*SP44*-*indC*) from day 1 to day 5 (Fig. 4A). The activity of each promoter was quantitatively compared using shaking-flask fermentation. The production of indigoidine in strain *S. albus*-*stnYp*-*indC* was approximately 6.1-, 4.1-, and 2.6-fold higher than the 5-day indigoidine accumulation in three strains *S. albus*-ermEp*-*indC*, *S. albus*-kasOp*-*indC*, and *S. albus*-*SP44*-*indC*, respectively (Fig. 4B). These results demonstrate that the promoter *stnYp* can not only efficiently activate the silent BGC of indigoidine but also significantly enhance the production of target metabolites.

### Engineering the multicistronic cassettes with promoter ***stnYp*** and achieving a high yield of aureonuclemycin and YM-216391 in ***Streptomyces*** hosts

**Fig. 5:**
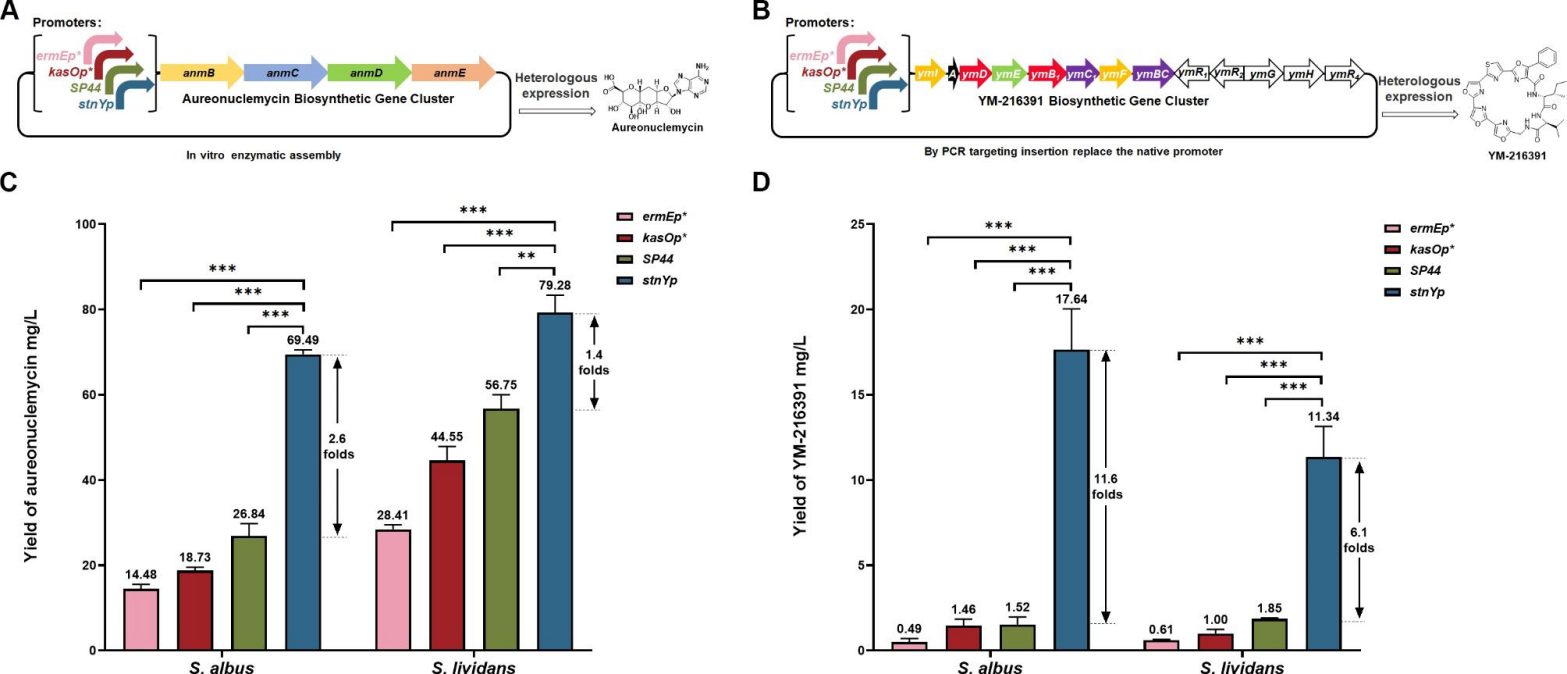
Applications of promoter *stnYp* for aureonuclemycin and YM-216391 overproduction in heterologous hosts. **(A)** Schematic for constructing recombinant strains in which different constitutive promoters (ermEp*, kasOp*, *SP44* and *stnYp*) were inserted upstream of the *anmB* gene. **(B)** Schematic for constructing recombinant strains in which different constitutive promoters (ermEp*, kasOp*, *SP44* and *stnYp*) were inserted upstream of the *ymI* gene. **(C)** Comparison of aureonuclemycin yield in recombinant strains with *anmBCDE* cassette driven by different promoters (ermEp*, kasOp*, *SP44* and *stnYp*) integrated into the genome of *S. albus* J1074 and *S. lividans* TK24. **(D)** Comparison of YM-216391 yield in recombinant strains with *ymI-BC* cassette driven by different promoters (ermEp*, kasOp*, *SP44* and *stnYp*) integrated into the genome of *S. albus* J1074 and *S. lividans* TK24. Statistical analyses were performed and marked (**, *p* ≤ 0.05; ***, *p* ≤ 0.01)

To demonstrate the applicability of the promoter *stnYp* in engineering natural product BGCs in multicistronic cassettes, the four-gene cluster for aureonuclemycin synthesis and the thirteen-gene BGC of YM-216391 were cloned under the promoter *stnYp*, and the production of both metabolites was detected. First, the *anmB-E* locus for aureonuclemycin synthesis under the promoter *stnYp* or other promoters (ermEp*, kasOp*, and *SP44*) was inserted into the integrative vector pSET152. All recombinant plasmids and the empty plasmid pSET152 were introduced into *S. albus* J1074 and *S. lividans* TK24, respectively (Fig. 5A). The BGC of YM-216391 contains 13 genes, in which the *ymI*-*ymBC* genes are arranged in the same orientation and appear tightly clustered. To assess whether the *ymI*-*ymBC* genes were co-transcribed, RT-PCR analyses were performed with specific primer pairs for the intergenic regions (*ymI*-*A*, *ymA*-*D*, *ymD*-*E*, *ymE*-*B1*, *ymB1*-*C1*, *ymC1*-*F*, and *ymF*-*BC*) (Additional file 1: Fig. S3). The results indicated that all the tested regions (*ymI*-*BC*) in the same direction were transcribed as an eight-gene multicistronic cassette, acting as an 8.5 kb operon. The promoter of the *ymI* gene may be responsible for the transcription of the *ymI-BC* cassette. To evaluate the performance of promoter substitution in the production of YM-216391, the native promoter of *ymI* in front of the YM-216391 biosynthetic cluster (cloned in cosmid pTG1104) was replaced by the promoter *stnYp* or three other promoters (ermEp*, kasOp*, *SP44*). The resulting plasmids YM-ermEp*, YM-kasOp*, YM-*SP44*, and YM-s*tnYp* were integrated into *S. albus* J1074 and *S. lividans* TK24 (Fig. 5B). The empty vector pJTU2554 was also transferred to *Streptomyces* species, which were used as negative controls.

Subsequently, the yields of aureonuclemycin and YM-216391 in the recombinant *S. albus* and *S. lividans* strains were determined using HPLC analysis. The fermentation results showed that the production of both aureonuclemycin and YM-216391 was significantly enhanced by gene clusters controlled by the promoter *stnYp* (Additional file 1: Fig. S4A, S4B, S5A, S5B). The yield of aureonuclemycin in strain *S. albus*-*stnYp*-aur reached 69.49 mg/L, approximately 4.8-, 3.7-, and 2.6-fold higher than those in *S. albus*-ermEp*-aur, *S. albus*-kasOp*-aur, and *S. albus*-*SP44*-aur, respectively (Fig. 5C). The yield of aureonuclemycin in *S. lividans*-*stnYp*-aur was 79.28 mg/L, approximately 2.8-, 1.8-, and 1.4-fold higher than those in *S. lividans*-ermEp*-aur, *S. lividans*-kasOp*-aur, and *S. lividans*-*SP44*-aur, respectively (Fig. 5C). These results demonstrate that the yield of aureonuclemycin was significantly enhanced by the promoter *stnYp*, which is more efficient than other reported promoters for high-output nucleoside antibiotics. Surprisingly, the production of YM-216391 in the engineered strain *S. albus*-*stnYp*-YM could reach 17.64 mg/L, while the yields in the other three engineered strains (*S. albus*-ermEp*-YM, *S. albus*-kasOp*-YM, and *S. albus*-*SP44*-YM) were only 0.49 mg/L, 1.46 mg/L, and 1.52 mg/L, respectively (Fig. 5D). The production of YM-216391 in *S. lividans*-*stnYp*-YM also reached 11.34 mg/L, which was significantly higher than that in the three engineered strains (*S. lividans*-ermEp*-YM, *S. lividans*-kasOp*-YM, and *S. lividans*-*SP44*-YM), at approximately 0.61 mg/L, 1.00 mg/L, and 1.85 mg/L, respectively (Fig. 5D). Therefore, both *S. albus*-*stnYp*-YM and *S. lividans*-*stnYp*-YM with the multicistronic cassette *ymI*-*BC* driven by the promoter *stnYp* exhibited the most significant enhancement in YM-216391 production, suggesting that the promoter *stnYp* was powerful and reliable in stimulating the expression of the multicistronic cassette.

Furthermore, the transcriptional levels of genes in the multicistronic cassette (*ymA*, *ymE*, *ymC1*, and *ymBC*) driven by different promoters in *S. albus* derivative strains were monitored at two-time points (24 and 72 h). The results showed that the transcriptional levels of *ymA*, *ymE*, *ymC1*, and *ymBC* driven by the promoter *stnYp* were significantly upregulated compared with those driven by ermEp*, kasOp*, and *SP44* (Additional file 1: Fig. S6A, S6B). The transcriptional level of the precursor peptide gene *ymA* at 24 h was strikingly improved under the control of the promoter *stnYp*, with 17.9-, 9.8-, and 8.1-fold higher than those controlled by the promoters ermEp*, kasOp*, and *SP44*, respectively (Additional file 1: Fig. S6A). As expected, the transcriptional variation in the BGCs was consistent with the improvement in the production of YM-216391.

### Engineering a tylosin A-producing ***S. fradiae*** strain with the promoter ***stnYp***

**Fig. 6:**
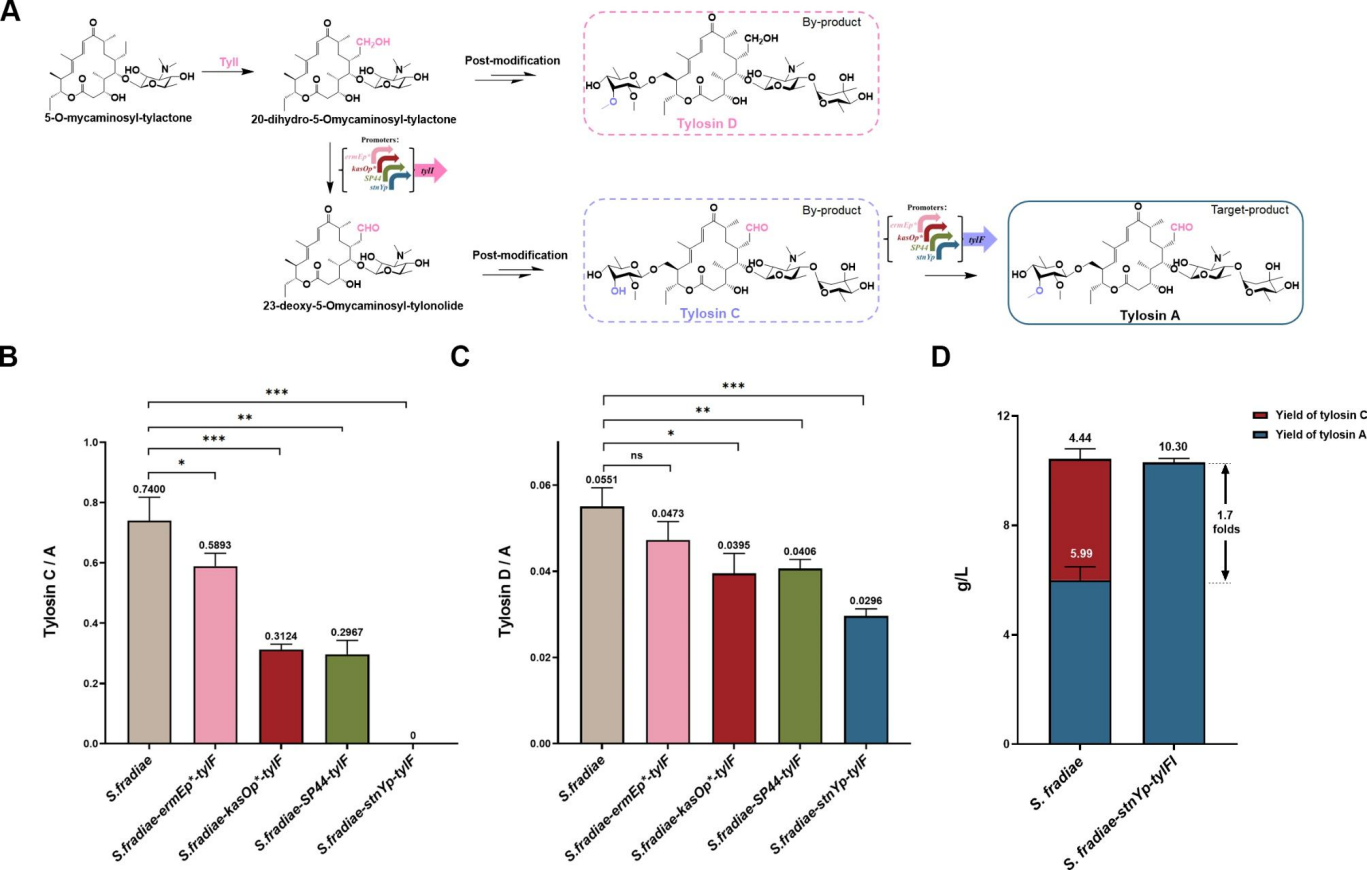
Application of promoter *stnYp* for by-products elimination of tylosin A in industrial *S. fradiae* strains. **(A)** Biosynthetic pathway of tylosin A. **(B)** The yield ratio of tylosin C/A in recombinant strains with *tylF* under the control of different promoters (ermEp*, kasOp*, *SP44* and *stnYp*). **(C)** The yield ratio of tylosin D/A in recombinant strains with *tylI* under the control of different promoters (ermEp*, kasOp*, *SP44* and *stnYp*). **(D)** Quantification of tylosin A yield in recombinant strains with *tylF* and *tylI* driven by promoter *stnYp*. Statistical analyses were performed and marked (*, *p* ≤ 0.1; **, *p* ≤ 0.05; ***, *p* ≤ 0.01)

Tylosin, a commercially available veterinary antibiotic produced by *S. fradiae*, consists of four structurally similar components (tylosin A, tylosin B, tylosin C, and tylosin D) [33]. The biological activity of the mixture is mainly attributed to tylosin A. For the tylosin A biosynthetic pathway, the final and rate-limiting step is O-methylation of the mycinose sugar moiety at the 3-position catalyzed by SAM-dependent O-methyltransferase TylF, of which insufficient expression leads to the accumulation of by-product tylosin C [34] (Fig. 6A). P450 TylI was proposed to catalyze successive steps of hydroxylation and alcohol oxidation at C-20 of 5-mycaminosyl-tylactone, and its insufficient expression may lead to the accumulation of another by-product, tylosin D [35] (Fig. 6A). Replacing native promoters with stronger promoters of the desired strength is a simple and efficient method to facilitate the conversion of intermediates into final products. To reduce the by-product tylosin C as much as possible, plasmids containing the gene *tylF* under the control of the promoters ermEp*, kasOp*, *SP44*, and *stnYp* were constructed and introduced into the industrial strain *S. fradiae*. The fermentation results detected by HPLC showed that the peak of tylosin C in the recombinant strain *S. fradiae*-*stnYp*-*tylF* completely disappeared, whereas those in other strains with *tylF* driven by ermEp*, kasOp*, or *SP44* were retained (Additional file 1: Fig. S7A). In the wild-type tylosin-producing strain, the titer of tylosin C/A was 0.740, and tylosin C/A was still 0.589, 0.312, and 0.297 in *S. fradiae* derivative strains, in which *tylF* was driven by the promoters ermEp*, kasOp*, and *SP44*, respectively. Only *S. fradiae stnYp-tylF* with *tylF* driven by the promoter *stnYp* completely converted tylosin C into tylosin A (Fig. 6B). Further, the conversion of shunt product tylosin D into tylosin A was achieved in the same way, with the insertion of different promoters upstream of the gene *tylI*, and the fermentation results detected using HPLC are shown in Figure S7B (Additional file 1). In the wild-type tylosin-producing strain, the tylosin D/A titer was 0.055. Overexpression of *tylI* with ermEp*, kasOp*, *SP44*, and *stnYp* reduced tylosin D production, resulting in titers of 0.047, 0.039, 0.041, and 0.030, respectively (Fig. 6C). Even if the strongest promoter, *stnYp*, could not completely convert tylosin D to tylosin A, the titer of tylosin D/A less than 0.035 met the requirements of industrial fermentation. Finally, *tylF* and *tylI* genes driven by the promoter *stnYp* were combined, and the titer of tylosin in the recombinant strain *S. fradiae*-*stnYp*-*tylFI* was determined (Additional file 1: Fig. S7C). The accumulation of tylosin A in this strain was approximately 10.30 g/L, 1.7-fold higher than that of the wild-type industrial strain (5.99 g/L) (Fig. 6D). These results demonstrated that substituting the promoter *stnYp* upstream of *tylF* and *tylI* genes can improve the bioconversion efficiency from tylosin C or tylosin D into tylosin A and increase the purity and yield of tylosin A.

## Discussion

*Streptomyces* are major producers of various natural products for both pharmaceutical and agricultural applications. However, under standard laboratory culture conditions, most biosynthetic genes for these metabolites are inactive or have very low expression levels. The preferred strategy for improving the productivity of natural products in *Streptomyces* is to enhance the transcription and expression of critical genes in natural product synthesis pathways. Strong and constitutive promoters are key to solving this problem and achieving the goal of natural product overproduction. In this study, a reliable constitutive promoter, *stnYp*, was identified and shown to be useful for multiple *Streptomyces* species, with higher activity and greater robustness than other widely used promoters (ermEp*, kasOp*, and *SP44*). It not only activates the transcription of a silent gene cluster (indigoidine gene cluster) but also enhances the expression of multicistronic biosynthetic cassettes (aureonuclemycin and YM-216391 gene clusters). Moreover, it can eliminate by-products and improve the production of tylosin A by the industrial strain. These findings provide a powerful tool for activating natural product biosynthesis pathways and engineering *Streptomyces* cell factories.

Bacterial promoters typically comprise a core promoter region and an upstream regulatory region [36]. The σ factor recognizes the core promoter region, which typically consists of two conserved motifs at nucleotide positions − 35 and − 10 relative to the TSS [37]. Based on the analysis of the TSS and TLS in the genome of *S. coelicolor* A3 and *S. lividans* TK24, the sequence of conserved − 10 motifs is TAnnnT, and the sequence of weakly conserved − 35 motifs is nTGACn [27, 28]. The core promoter region of *stnYp* is TAGCAT in the − 10 motifs and TTGGCG in the − 35 motifs, which is quite similar to the consensus sequence recognized by the σ factor HrdB in *Streptomyces* [30]. In addition to the core promoter region, promoters usually contain other *cis*-regulatory elements upstream of the − 35 motifs for transcription factor binding, which play a relevant role in gene expression regulation. In general, there are three mechanisms of transcriptional activators: Class I activation, Class II activation, and activation by conformational change [31, 36]. Activators in the Class I activation mechanism bind to the upstream regulatory regions and recruit RNAP to the promoter by interacting directly with the αCTD of RNAP [38]. The linker joining the αCTD and the α-subunit N-terminal domain of RNAP is flexible, and the activators can bind to multiple sites in the upstream region of promoters. The best example of activators functioning in the Class I activation mechanism is the action of the cyclic AMP receptor protein at the *lac* promoter [39]. In addition, in *E. coli*, the RNAP αCTD can recognize AT-rich regions (UP elements, from − 60 bp to –40 bp) upstream of the − 35 motifs in some promoters to enhance transcription. For example, the UP element of the promoter *rrnB* P1 for the expression of rRNA operons can increase transcription levels approximately 30-fold in vivo, where an AT-rich sequence is favorable for interaction [40]. The region adjacent to the UP element (from − 60 bp to –150 bp) contains the binding sites for activator protein Fis, further increasing the approximately 10-fold activity of promoter *rrnB* P1 [41]. The results of this study showed that the promoter *stnYp* contains a GC-rich region upstream of the − 35 motifs, and the 60 bp region upstream of the − 35 motifs of this promoter is important for its transcription efficiency. Regarding transcriptional activity, the identified promoter regions of *stnYp* appeared to be stable and suitable for heterologous gene expression in various *Streptomyces* strains. This implies that *cis*-acting elements may exist within the upstream element of the promoter *stnYp*, contributing to transcription enhancement, which requires further investigation.

Although thousands of bioactive metabolites have been isolated from various *Streptomyces* strains, most have not yet been commercialized. The main reason for this is that the yield of fermentation metabolites is too low to achieve economical large-scale production. The synthetic pathways for natural products are tightly regulated and often require complex physiological and environmental signals to induce their expression [42]. Random mutagenesis and fermentation optimization are effective strategies for increasing the yield of microbial products, but these methods are time-consuming and labor-intensive [43]. Promoter engineering within BGCs is perhaps the most effective and widely used strategy for improving biosynthetic performance. Replacing native inefficient promoters with well-characterized, strong constitutive promoters can eliminate the influence of intrinsic regulatory systems and activate the expression of silent BGCs [14]. The activation or upregulation of secondary metabolite biosynthetic genes by strong constitutive promoters is a simple and powerful strategy for discovering new bioactive natural products. Constitutive promoter substitution and heterologous gene expression are effective metabolic engineering strategies that have been widely used to improve the yield, purity, and stability of industrial strains to produce target metabolites [8]. In this study, the overproduction of indigoidine (natural product of silent BGCs), aureonuclemycin, and YM-216391 (natural product of BGCs in multicistronic cassettes) was achieved as their BGCs were under the control of the promoter *stnYp* in various strains, which were significantly higher than those with other constitutive promoters (ermEp*, kasOp*, and *SP44*) frequently used in research and industry. These findings demonstrated that the promoter *stnYp* is a strong constitutive promoter with high robustness, wide suitability, and great application potential.

The cyclic peptide YM-216391 belongs to a family of ribosomally synthesized and post-translationally modified peptides (RiPPs) isolated as cytotoxic compound from *S. nobilis* JCM 4274. YM-216391 exhibits remarkable antitumor activity and inhibits the growth of human cervical cancer cells [44]. In its biosynthetic pathway, a precursor peptide (YmA) undergoes a series of complex modifications catalyzed by enzymes encoded in the YM-216391 gene cluster. By modifying the amino acid composition of the precursor peptide, researchers have generated a series of analogs to develop anticancer drug candidates [44]. Given that the precursor peptide is the substrate for the biosynthetic pathway, a sufficient precursor peptide enables the overproduction of RiPPs and results in a high yield of YM-216391. Therefore, improving the production of YM-216391 is vital for the subsequent combinatorial biosynthesis of new bioactive compounds. Previous studies have shown that heterologous expression of wild-type BGC in *S. lividans* reached an approximately 0.16 mg/L yield of YM-216391 [45]. Inactivating the negative regulator gene enhanced the production of YM-216391 to 3.84 mg/L [45]. Assisted by a multiplexed site-specific integrated super host, the integration of four copies of the BGCs of YM-216391 resulted in a significant elevation in yield (36.4 mg/L in *S. coelicolor* M1452 and 12.1 mg/L in *S. coelicolor* M1446) [46]. Notably, *S. coelicolor* M1452 carries a point mutation [S433L] in *rpoB* (encoding the RNAP β-subunit), leading to the overproduction of secondary metabolites [47, 48]. In this study, YM-216391 production reached 17.64 mg/L by overexpressing one copy of the *ymI-BC* multicistronic cassette via the promoter *stnYp* in *S. albus* J1074. Therefore, combining the strong constitutive promoter with super-inclusive hosts through metabolic engineering strategies is conducive to further production improvement of biosynthetic performance for the target natural products.

In the biosynthetic pathways of natural products, insufficient amounts or ineffective activity of certain rate-limiting enzymes often leads to the accumulation of nonspecific intermediates. The less active components produced through biosynthesis compete with the most active components for substrates and energy, resulting in a low yield and deficient purity of the target products. Thus, tuning the expression of biosynthetic genes is important for reducing the output of undesired compounds and enhancing the production of the desired compounds. Overexpression of rate-limiting genes with strong promoters can accelerate the conversion of intermediates into final products. Eliminating the production of by-products is vital for enhancing the titer of the desired compounds. A polyketide assembly line often produces multiple structurally related compounds, such as tylosin [49], erythromycin [50] and spinosad [51]. Tylosin exhibits broad-spectrum antimicrobial activity against most gram-positive bacteria and gram-negative pathogens. It exerts its antibacterial activity by binding the 23S rRNA in the 50S subunit of the bacterial ribosome; hence, it is widely used in animal husbandry because of its broad-spectrum antimicrobial activity, obvious growth promotion effect, and lack of cross-resistance with humans [33]. Because tylosin is a pharmaceutical product, its quality (the number of impurities and their content) must be strictly controlled to ensure compliance with all regulatory guidelines [49]. Overexpression of the genes encoding bottleneck enzymes in the tylosin biosynthetic pathway using the promoter *stnYp* in the *S. fradiae* industrial strain resulted in a 1.7-fold increase in the yield of tylosin A (10.30 ± 0.12 g/L). This implies that *stnYp* is applicable for promoting the production and purity of natural products in industrial strains. The *S. fradiae-stnYp-tylF* strain, which overexpresses the *tylF* gene driven by *stnYp*, converts tylosin C completely to tylosin A, suggesting that there is sufficient SAM-dependent O-methyltransferase for tylosin A generation. However, substituting the promoter *stnYp* did not completely convert tylosin D to tylosin A. This may be the result of the weak reduction activity of tylosin reductase in cells, probably reducing a small amount of tylosin A to tylosin D. Tylosin reductase exhibits broad substrate specificity for many macrolide aldehydes (as normal and shunt metabolites of tylosin biosynthesis), and the physiological role of macrolide detoxification for bacteria is closely related to bacterial growth [52]. In any case, the successful use of the promoter *stnYp* in the overproduction of tylosin has great application potential in industrial strains to improve the yield and purity of bioactive metabolites with similar structures.

## Conclusions

A new constitutive promoter, *stnYp*, has been identified, and its activity is much higher than those of the three widely used promoters. The promoter *stnYp* is robust and adaptable for expressing biosynthetic genes in different host strains of five *Streptomyces* species, namely *S. albus* J1074, *S. coelicolor* M1154, *S. lividans* TK24, *S. venezuelae* ISP5230, and *S. fradiae*. Furthermore, the promoter *stnYp* exhibits efficient gene activation, consistent heterologous expression, and flexible host compatibility. In addition, the promoter *stnYp* enhances the production of diverse natural products encoded by different BGCs in hosts with distinct genetic backgrounds. Finally, the promoter *stnYp* can be useful in synthetic biology to activate cryptic biosynthetic clusters and optimize natural product biosynthesis pathways in *Streptomyces* species.

## Materials and methods

### Bacterial strains, media and growth conditions

All the bacterial strains and plasmids used in this study are listed in the Supporting Information Table S1 and S2 (Additional file 1). Luria–Bertani (LB) broth and plates were used for the cultivation of *Escherichia coli* strains. All *Streptomyces* strains were cultivated in the malt extract–yeast extract–maltose (YEME) medium for liquid inoculation. Strain *E. coli* DH5α was used for general cloning cultivated in LB broth at 37 °C. Strain *E. coli* ET12567 containing pUZ8002 was used for introducing plasmids from *E. coli* into *Streptomyces* by intergeneric conjugation cultivated in LB broth at 37 °C. Strain *E. coli* BW25113 containing pIJ790 was used for the construction of recombinant plasmids via *λ*-Red mediated recombination cultivated in LB broth at 30 °C. For spore preparations, strains *S. albus* J1074, *S. lividans* TK24, *S. coelicolor* M1154 and their derivatives were cultivated on mannitol soya flour (MS) agar plates. Strain *S. venezuelae* ISP5230 and its derivatives were cultivated on maltose-yeast extract-malt extract (MYM) agar plates. The conjugation of all *Streptomyces* strains was maintained on MS agar plates. All the operations of *Streptomyces* strains were carried out at 30 °C. When necessary, the media were supplemented with antibiotics at the following concentrations: kanamycin (50 µg/mL), chloramphenicol (25 µg/mL), apramycin, (50 µg/mL), ampicillin (100 µg/mL), nalidixic acid (25 µg/mL).

### Construction of recombinant plasmids and strains

To construct the *xylE* reporter plasmids and the *Streptomyces* derivatives, the promoter fragments of different lengths (*stnYp*_*339*_, *stnYp*_*up150*_, *stnYp*_*up100*_, *stnYp*_*up80*,_*stnYp*_*up60*_, *stnYp*_*up30*_, and *stnYp*_*up10*_) were PCR-amplified from the genome of *S. flocculus* CGMCC4.1223 with the corresponding primer pairs. All the primers used in this study are listed in the Supporting Information Table S3 (Additional file 1). The fragment of promoter ermEp* was amplified from pIB139, the fragment of promoter kasOp* was amplified from the genome of *S. coelicolor* and the fragment of promoter *SP44* was synthesized. These promoter fragments were treated with the endonucleases *Bam HI* and *Bcu I*, and ligated with the same treated plasmid pDR3 harboring *xylE* gene, generating the corresponding recombinant plasmids pDR3-*stnYp*_*339*_, pDR3-*stnYp*_*up15*0_, *pD*R3-*stnYp*_*up100*_, pDR3*-stnYp*_*up80*_, pDR3-*stnYp*_*up60*_, pDR3-*stnYp*_*up30*_, pDR3-*stnYp*_*up10*_, pDR3-ermEp*, pDR3-kasOp* and pDR3-*SP44*, respectively (Table S2) (Additional file 1). These recombinant plasmids were then introduced into *Streptomyces* and integrated at the phage *Φ31* attachment site (*attB*) in genome forming the derivative strains (Table S1) (Additional file 1).

To construct the plasmids or strains containing *indC* gene under the control of different promoters, the full length of the *indC* fragment was PCR-amplified from the genome of *S. albus* J1074 and cloned into plasmid pSET152 between the sites of *Bam HI* and *Xba I*, generating plasmid pSET-*indC*. The fragment of the promoter *stnYp* were amplified from the genome of *S. flocculus* CGMCC4.1223 and inserted into plasmid pSET-*indC* at the site of *Bcu I*, forming plasmid pSET-*stnYp*-*indC*. The plasmids containing *indC* gene under other promoters were also constructed in the same way. These recombinant plasmids were then introduced into *Streptomyces* respectively, and integrated at the phage *Φ31* attachment site (*attB*) in genome to generate corresponding derivative strains (*S. albus*-*stnYp*-*indC*, *S. albus*-ermEp*-*indC*, *S. albus*-kasOp*-*indC*, *S. albus*-*SP44*-*indC* and *S. albus*-pSET152) (Table S1) (Additional file 1).

To construct the aureonuclemycin overexpressing plasmids and the corresponding *Streptomyces* derivatives, the fragments of the multicistronic cassette (*aurBCDE*) were amplified from pSET-*anmBCDE* and the fragment of the promoter *stnYp* was amplified with primer pairs of 152YF/aurYR (Table S3) (Additional file 1). The two fragments were ligated into plasmid pSET152 treated with *Bam HI* and *Xba I* endonucleases, generating plasmid pSET-*stnYp*-*anmBCDE*. Similarly, the plasmids containing the multicistronic cassette (*aurBCDE*) under the control of other promoters were constructed. The corresponding *Streptomyces* derivatives (*S. albus*-ermEp*-aur, *S. albus*-kasOp*-aur, *S. albus*-*SP44*-aur, *S. albus*-*stnYp*-aur, *S. lividans*-ermEp*-aur, *S. lividans*-kasOp*-aur, *S. lividans*-*SP44*-aur, *S. lividans*-*stnYp*-aur and *S. lividans*-pSET152) were further constructed by genomic integration.

To construct the YM-216391 overexpressing plasmids and the corresponding *Streptomyces* derivatives, the cosmid pTG1104 (the BGC of YM-216391) was introduced into strain *E. coli* BW25113/pIJ790 (Table S2) (Additional file 1). The fragment *neo* cassette-*stnYp* was obtained by overlapping- PCR amplification of kanamycin resistance gene cassette *neo*-*oriT* amplified from pJTU4659 and the promoter *stnYp*, which was used to replace the native promoter of *ymI* using PCR targeting and *λ*-Red-mediated recombination [53] (Table S3) (Additional file 1). Similarly, the plasmids containing the BGC of YM-216391 in the control of other promoters were constructed. The corresponding *Streptomyces* derivatives (*S. albus*-ermEp*-YM, *S. albus*-kasOp*-YM, *S. albus*-*SP44*-YM, *S. albus*-*stnYp*-YM, *S. albus*-pJTU2554, *S. lividans*-ermEp*-YM, *S. lividans*-kasOp*-YM, *S. lividans*-*SP44*-YM, *S. lividans*-*stnYp*-YM and *S. lividans*-pJTU2554) were further constructed by genome integration.

To construct the plasmids or strains containing the *tylF* gene under the control of different promoters, the gene *tylF* encoding SAM-dependent O-methyltransferases was amplified from genome of *S. fradiae* and the promoter *stnYp* were amplified by PCR from the genome of *S. flocculus* CGMCC4.1223 and cloned into plasmid pSET152 between the site of *Bam HI*, generating plasmid pSET-*stnYp*-*tylF*. The plasmids containing the *tylF* gene under other promoters were also constructed in the same way. These recombinant plasmids were then introduced into *Streptomyces* respectively, and integrated at the phage *Φ31* attachment site (*attB*) in genome to generate corresponding derivative strains (*S. fradiae*-ermEp*-*tylF*, *S. fradiae*-kasOp*-*tylF*, *S. fradiae*-*SP44*-*tylF* and *S. fradiae*-*stnYp*-*tylF*) (Table S1) (Additional file 1).

To construct the plasmids or strains containing the *tylI* gene under the control of different promoters, the gene *tylI* encoding P450 was amplified from the genome of *S. fradiae* and the promoter *stnYp* were amplified by PCR from the genome of *S. flocculus* CGMCC4.1223 and cloned into plasmid pSET152 between the site of *Bam HI*, generating plasmid pSET-*stnYp*-*tylI*. The plasmids containing the *tylI* gene under other promoters were also constructed in the same way. These recombinant plasmids were then introduced into *Streptomyces* respectively, and integrated at the phage *Φ31* attachment site (*attB*) in genome to generate corresponding derivative strains (*S. fradiae*-ermEp*-*tylI*, *S. fradiae*-kasOp*-*tylI*, *S. fradiae*-*SP44*-*tylI* and *S. fradiae*-*stnYp*-*tylI*) (Table S1) (Additional file 1).

To construct the plasmids or strains containing combined overexpression the *tylF* and *tylI* genes under the control of different promoters, the gene *tylF* was amplified from the genome of *S. fradiae* and cloned into plasmid pSET-*stnYp*-*tylI* between the site of *Bcu I*, generating plasmid pSET-*stnYp*-*tylFI*. The recombinant plasmid was then introduced into *Streptomyces* and integrated at the phage *Φ31* attachment site (*attB*) in genome to generate corresponding derivative strains (*S. fradiae*-*stnYp*-*tylFI*) (Table S1) (Additional file 1).

### RNA extraction and quantitative RT-PCR experiment

Mycelia of *S. albus* derivatives grown at 30℃ in YEME media were harvested at different time points (24 h, 48 and 72 h) and immediately frozen for RNA extraction using the bacterial RNA Kit purchased from Tiangen (Beijing, China) according to the manufacturer’s instructions. Genomic DNA was removed with gDNA Eraser (TaKaRa, Dalian, China) and checked by PCR to eliminate the possibility of DNA contamination. First-strand cDNA was synthesized using the PrimeScript RT reagent kit (TaKaRa, Dalian, China) according to the manufacturer’s protocol.

For RT-PCR analyses, cDNA was used as a template for PCR reactions using Taq polymerase and specific primers for the regions to test. The amplification conditions were as follows: initial denaturation at 95℃ for 5 min followed by 35 cycles of 95℃ for 30 s, 60℃ for 30 s and 72℃ for 30 s, with a final extension step at 72℃ for 5 min. The resulting RT-PCR products were separated in 1% agarose gels and stained with ethidium bromide for visualization.

For qRT-PCR analyses, the cDNA was used as template in quantitative reactions with TB Green Premix Ex Taq II (TaKaRa, Dalian, China) following the manufacturer’s instructions. The reactions were run in ABI 7500Fast and the amplification protocol was a 2-step cycling PCR program: 1 cycle at 95℃ for 30s followed by 40 cycles of 5s at 95℃ and 34 s at 60℃. An additional melting curve step was used at the end of the reaction to assess the specificity of the amplified products. The sigma factor gene *hrdB* was used as an internal reference for qRT-PCR normalization. Each qRT-PCR was set with three parallels and five independent experiments were performed. The relative transcription level of each gene was evaluated using the 2^-ΔΔCt^ method.

### 5’-RACE Experiments

The TSS were identified using a 5’-RACE system for rapid amplification of cDNA ends (Roche, CHE), using the manufacturer’s instructions (version 2.0). 1 µg of total RNA was extracted from the culture of *S. flocculus* CGMCC4.1223 harvested after 36 h of growth at 30℃ and was used to carry out cDNA synthesis with specific primers. The cDNA was purified and treated with terminal deoxynucleotidyl transferase (TdT) to add poly(dC) tails to its 5’ends. After an initial PCR amplification of the tailed fragments with the 5’-RACE abridged anchor primer and subsequent amplifications with the universal amplification primer and specific nested primers, the defined amplification products were observed. These amplified products were purified and sequenced to analyze the position of the TSS.

### Assessment of promoter strength with XylE as reporter

Quantitative measurements of the XylE (catechol-2,3-dioxygenase) activity were performed essentially as previously described [18]. Briefly, 1 mL of the cell culture at different time points were harvested and centrifuged at 4000 rpm at 4℃ for 10 min to remove the supernatant. Cells were washed with cold deionized water three times and resuspended in 1 mL of sample buffer (100 mM phosphate buffer, pH 7.5; 20 mM Na-EDTA, pH 8.0; 10% v/v acetone) followed by sonication on ice. After adding 0.1% Triton X-100, the samples were incubated on ice for 10 min and centrifuged at 12,000 rpm at 4℃ for 10 min. The cell lysates were transferred into fresh tubes, and 1 mL assay buffer (10 mM phosphate buffer, pH 7.5; 0.2 mM catechol 2,3-dioxygenase) was added into 20 µL of the cell lysate. The change in absorbance at 375 nm (A375) was monitored and the XylE activity was calculated as the rate of change in optical density at 375 nm per minute per milligram of protein.

### Measurement of indigoidine production

The measurement of the indigoidine production was performed as previously described [54]. Briefly, *S. albus* J1074 and its derivatives were germinated in 30 mL of YEME for 24–30 h (220 r, 30℃) as seed culture, and then 0.5 mL of each seed culture was transferred to 50 mL R5A liquid medium. The fermentation samples were harvested at 1, 2, 3, 4 and 5 days, and centrifuged at 12,000 rpm for 10 min. Then, 100 µl of the supernatant was diluted with 900 µl of dimethyl sulfoxide (DMSO) and was used to measure at OD_600_. All experiments were performed in triplicate.

### Measurement of aureonuclemycin production

The measurement of the Aureonuclemycin production was performed as previously described [55]. Briefly, *S. albus* and *S. lividans* and their derivatives were germinated in 30 mL of YEME for 24–30 h (220 r, 30℃) as a seed culture, and then 1mL of each seed culture was transferred to 50mL fermentation medium (dextrin 4%, tomato paste 0.75%, NZ amine A 0.25%, yeast extract 0.5%, pH = 7.0), and incubated at 30 °C, 220 r for 4 days. The fermentation cultures were sampled and centrifuged at 12,000 rpm for 10 min; and the supernatants were used to analyze aureonuclemycin production by HPLC (1260 series, Agilent, USA) using a reverse-phase column (ACE Excel, C18, 5 μm, 4.6 × 150 mm) with UV detection at 260 nm under the following program, a water (supplemented with 0.1% formic acid): methanol gradient was used as the mobile phase: 0–25 min, 5-100% methanol; 25–30 min, 100% methanol; 30–31 min, 100-5% methanol; 31–40 min, 5% methanol. The flow rate was 0.6 mL/min and aureonuclemycin eluted at approximately 16 min.

### Measurement of YM-216391 production

The measurement of the YM-216391 production was performed as previously described [44]. Briefly, *S. albus* and *S. lividans* and their derivatives were germinated in 30 mL of YEME for 24–30 h at 30 °C as a seed culture, and then 1 mL of each seed culture was transferred to 50 mL fermentation medium (yeast 0.4%, malt extract 1%, glucose 0.4%, pH7.2), and incubated at 30 °C, 220 r for 7 days. The fermentation broth was extracted twice with ethyl acetate, the organic layer was concentrated in vacuo and then the residue was dissolved in methanol for analysis by HPLC (1260 series, Agilent, USA) using a reverse-phase column (ACE Excel, C18, 5 μm, 4.6 × 150 mm) with UV detection at 287 nm under the following program: 0–4 min, 5% acetonitrile; 4–10 min, 5-20% acetonitrile; 10–20 min, 20-100% acetonitrile; 20–25 min, 100% acetonitrile; 25–26 min, 100-5% acetonitrile; 26–35 min, 5% acetonitrile. The flow rate was 0.6 mL/min and YM-216391 eluted at approximately 19 min.

### Measurement of tylosin production

The measurement of the Tylosin production was performed as previously described [56]. Briefly, *S. fradiae* and its derivatives were germinated in 30 mL of seed medium (soybean meal 0.5%, yeast extract 0.3%, corn steep liquor 1.0%, calcium carbonate 0.2% and soybean oil 0.5%, pH = 7.0-7.2) at 30℃, 220 r for 2 days, and then 1.5 mL of seed culture was transferred to 30 mL fermentation medium (corn powder 1.0%, corn protein 0.6%, fish meal 0.7%, calcium carbonate 0.3%, NaCl 0.05%, soybean oil 4%, betaine HCl 0.5%, CoCl_2_ 0.0001%, pH 7.0-7.2) and incubated at 30 °C, 220 r for 7 days. The fermentation samples were centrifuged at 12,000 rpm for 10 min and the supernatants after being diluted 20-fold were used to directly analyze tylosin production by HPLC (1260 series, Agilent, USA) using a reverse-phase column (ACE Excel, C18, 5 μm, 4.6 × 150 mm) with UV detection at 280 nm under the following program, water (supplemented with 0.1% formic acid): acetonitrile gradient was used as the mobile phase: 0–4 min, 25% acetonitrile; 4–26 min, 25-40% acetonitrile; 26–27 min, 40-100% acetonitrile; 27–32 min, 100% acetonitrile; 32–33 min, 100-25% acetonitrile; 33–40 min, 25% acetonitrile. The flow rate was 0.6 mL/min, tylosin A eluted at approximately 20 min, tylosin C eluted at approximately 15 min, and tylosin D eluted at approximately 18 min.

### Statistical analysis

All experiments were performed at least three times and the results were presented as mean ± standard deviation (SD) values. Statistical analysis was performed with Student’s t test. (*p* ≤ 0.1 was considered significant difference marked with a “*”, *p* ≤ 0.05 was considered extremely significant difference marked with a “**”, *p* ≤ 0.01 was considered extremely significant difference marked with a “***”).

## Electronic supplementary material

Below is the link to the electronic supplementary material.

Additional file 1: Figure S1. Transcription analyses of *xylE* in the control of different promoters; **Figure S2**. Nucleotide sequences of promoter *stnYp*_*339*_; **Figure S3**. The co-transcription analysis of genes in the *ym* cluster; **Figure S4**. Applications of promoter *stnYp* for aureonuclemycin overproduction in heterologous hosts; **Figure S5**. Applications of Promoter *stnYp* for YM-216391 overproduction in heterologous hosts; **Figure S6**. Transcription analyses of YM-216391 biosynthetic gene cluster; **Figure S7**. Applications of promoter *stnYp* for eliminating by-products of tylosin in industrial strains *S. fradiae*; Table S1. Bacterial strains used in this study; **Table S2**. Plasmids used in this study; **Table S3**. Primers used in this study.

## Data Availability

All data for this study are included in this published article and its additional file.
