# Supplementary material for: Identification and characterization of a strong constitutive promoter stnYp for activating biosynthetic genes and producing natural products in streptomyces
Source: Microb Cell Fact. 2023 Jul 13;22:127. doi: 10.1186/s12934-023-02136-9 (PMC10339500; doi:10.1186/s12934-023-02136-9)
Supplement: Supplementary file 1 — Additional file 1: Figure S1. Transcription analyses of xylE in the control of different promoters; Figure S2. Nucleotide sequences of promoter stnYp339; Figure S3. The co-transcription analysis of genes in the ym cluster; Figure S4. Applications of promoter stnYp for aureonuclemycin overproduction in heterologous hosts; Figure S5. Applications of Promoter stnYp for YM-216391 overproduction in heterologous hosts; Figure S6. Transcription analyses of YM-216391 biosynthetic gene cluster; Figure S7. Applications of promoter stnYp for eliminating by-products of tylosin in industrial strains S. fradiae; Table S1. Bacterial strains used in this study; Table S2. Plasmids used in this study; Table S3. Primers used in this study. [file 12934_2023_2136_MOESM1_ESM.pdf]

Additional file 1

Identification and characterization of a strong constitutive promoter *stnYp* for activation of biosynthetic genes and production of natural products in *Streptomyces*

Wenli Guo[1], Zhihong Xiao[1], Tingting Huang[1,3], Kai Zhang[4], Hai-Xue Pan[4], Gong-Li Tang[4], Zixin Deng[1,3], Rubing Liang[1,3]*, and Shuangjun Lin[1,2,3]*

[1] State Key Laboratory of Microbial Metabolism, Joint International Research Laboratory on Metabolic & Developmental Sciences, School of Life Sciences & Biotechnology, Shanghai Jiao Tong University, 800 Dongchuan Road, Shanghai 200240, China

[2] Frontiers Science Center for Transformative Molecules, Shanghai Jiao Tong University, 800 Dongchuan Road, Shanghai 200240, China

[3] Haihe Laboratory of Synthetic Biology, Tianjin 300308, China

[4] State Key Laboratory of Bioorganic and Natural Products Chemistry, Center for Excellence in Molecular Synthesis, Shanghai Institute of Organic Chemistry, University of Chinese Academy of Sciences, Chinese Academy of Sciences, 345 Lingling Road, Shanghai 200032, China

Corresponding author:

Rubing Liang: State Key Laboratory of Microbial Metabolism, Joint International Research Laboratory on Metabolic & Developmental Sciences, School of Life Sciences & Biotechnology, Shanghai Jiao Tong University, 800 Dongchuan Road, Shanghai 200240, China. Email: icelike@sjtu.edu.cn

Shuangjun Lin: State Key Laboratory of Microbial Metabolism, Joint International Research Laboratory on Metabolic & Developmental Sciences, School of Life Sciences & Biotechnology, Shanghai Jiao Tong University, Shanghai 200240, China; Frontiers Science Center for Transformative Molecules, Shanghai Jiao Tong University, 800 Dongchuan Road, Shanghai 200240, China. Email: linsj@sjtu.edu.cn

Figure S1 Transcription analyses of *xylE* in the control of different promoters.

Figure S2 Nucleotide sequences of promoter *stnYp*$_{339}$.

Figure S3 The co-transcription analysis of genes in the *ym* cluster.

Figure S4 Applications of promoter *stnYp* for aureonuclemycin overproduction in heterologous hosts.

Figure S5 Applications of Promoter *stnYp* for YM-216391 overproduction in heterologous hosts.

Figure S6 Transcript assay of YM-216391 biosynthetic gene cluster.

Figure S7 Applications of promoter *stnYp* for eliminating by-products of tylosin in industrial strains *S. fradiae*.

Table S1. Bacterial strains used in this study.

Table S2. Plasmids used in this study.

Table S3. Primers used in this study.

Figure S1. Transcription analyses of *xylE* in the control of different promoters. Quantitative analysis of the transcriptional levels of *xylE* reporter gene driven by different promoters in *S. albus* J1074 after 24 h, 48 h and 72 h culture in YEME. Transcription of *hrdB* gene was used to normalize the transcription levels in different samples. The relative transcription level of *ermEp** were set as 1.0.

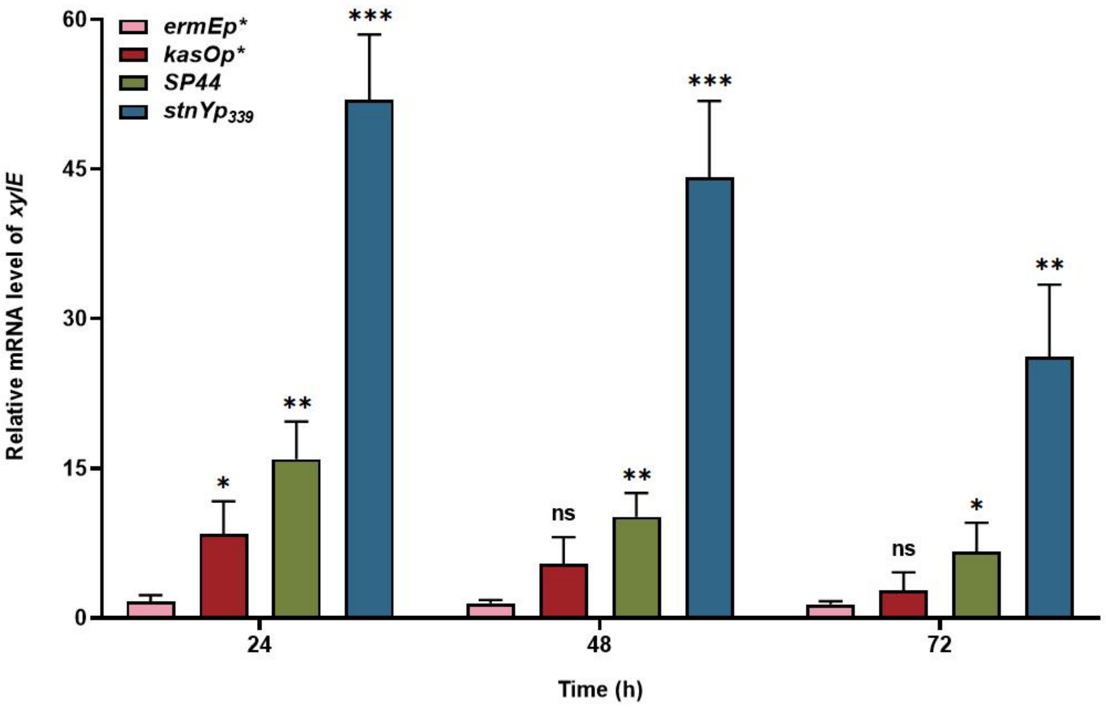

Figure S2. Nucleotide sequences of promoter *stnYp*$_{339}$. The sequence is numbered on the left. The TSS is indicated by a bent arrow and bigger letters. The TSS was determined by rapid amplification of 5'-cDNA ends (5'RACE). The putative −10 and −35 motifs of *stnYp*$_{339}$ are marked by dashed frames. Promoter elements of the *stnYp*$_{339}$ were deduced based on promoter prediction database SAPPHIRE. The translation start codon of *stnY* gene is marked in gray letters and the translated amino acids are given below the nucleotide sequence. TSS, Transcription Start Site.

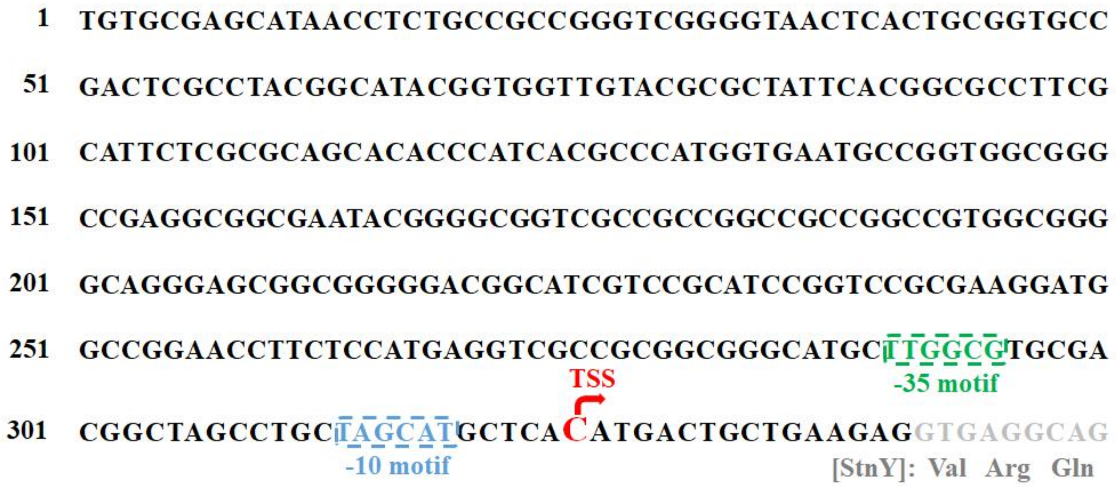

Figure S3. The co-transcription analysis of genes in the *ym* cluster. Horizontal black arrows above genes represent the putative transcripts, namely, *ymI-BC*. The present products of the intergenic regions showed that the two genes are co-transcribed, otherwise their transcripts were independent. a: the cosmid pTG1104 was used as a template. b: the cDNA of *S. albus*-pTG1104 was used as a template.

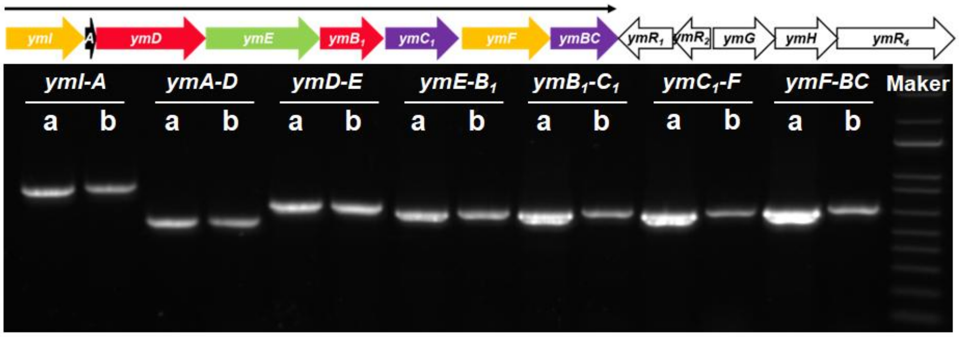

Figure S4. Applications of promoter *stnYp* for aureonuclemycin overproduction in heterologous hosts. (A) Chromatograms of aureonuclemycin production in five recombinant strains (*S. albus*-*ermEp**-aur, *S. albus*-*kasOp**-aur, *S. albus*-*SP44*-aur, *S. albus*-*stnYp*-aur and *S. albus*-pSET152). (B) Chromatograms of aureonuclemycin production in five recombinant strains (*S. lividans*-*ermEp**-aur, *S. lividans*-*kasOp**-aur, *S. lividans*-*SP44*-aur, *S. lividans*-*stnYp*-aur and *S. lividans*-pSET152).

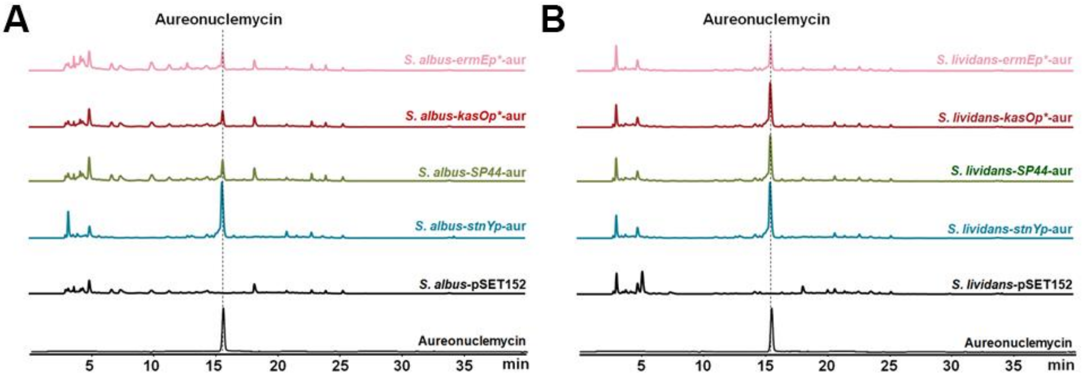

Figure S5. Applications of Promoter *stnYp* for YM-216391 overproduction in heterologous hosts. (A) Chromatograms of YM-216391 production in five recombinant strains (*S. albus-ermEp**-YM, *S. albus-kasOp**-YM, *S. albus-SP44*-YM, *S. albus-stnYp*-YM and *S. albus*-pJTU2554). (B) Chromatograms of YM-216391 production in in five recombinant strains (*S. lividans-ermEp**-YM, *S. lividans-kasOp**-YM, *S. lividans-SP44*-YM, *S. lividans-stnYp*-YM and *S. lividans*-pJTU2554).

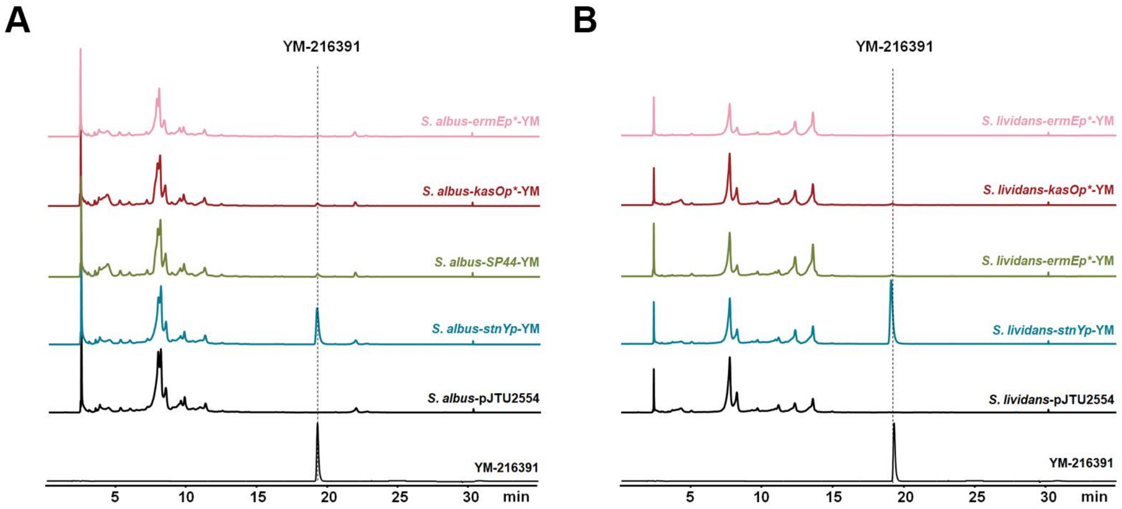

Figure S6. Transcript assay of YM-216391 biosynthetic gene cluster. The transcription of representative *ym* genes in the four recombination strains were detected at 24 h and 72 h. Transcription of *hrdB* gene was used to normalize the transcription levels in different samples. The relative transcription level of *ym* genes under the control of promoter *ermEp** was valued to 1.0.

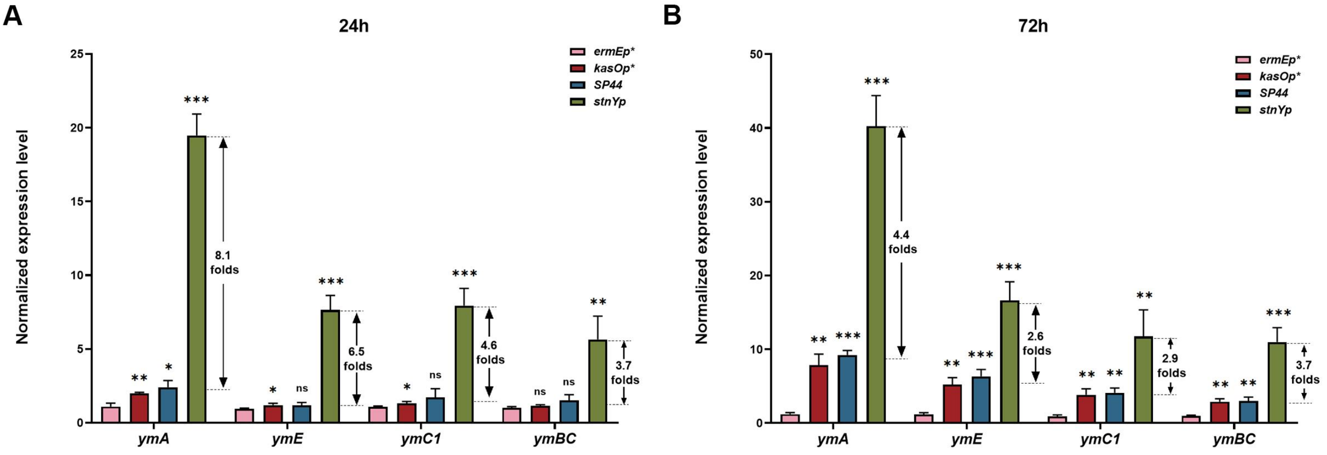

Figure S7 Applications of promoter *stnYp* for eliminating by-products of tylosin in industrial strains *S. fradiae*. (A) Chromatograms of fermentation culture of the recombinant strains with *tylF* driven by promoters *ermEp**, *kasOp**, *SP44* or *stnYp*. (B) Chromatograms of fermentation culture of the recombinant strains with *tylI* driven by promoters *ermEp**, *kasOp**, *SP44* or *stnYp*. (C) Chromatograms of fermentation culture of the recombinant strains with *tylF* and *tylI* driven by promoter *stnYp*.

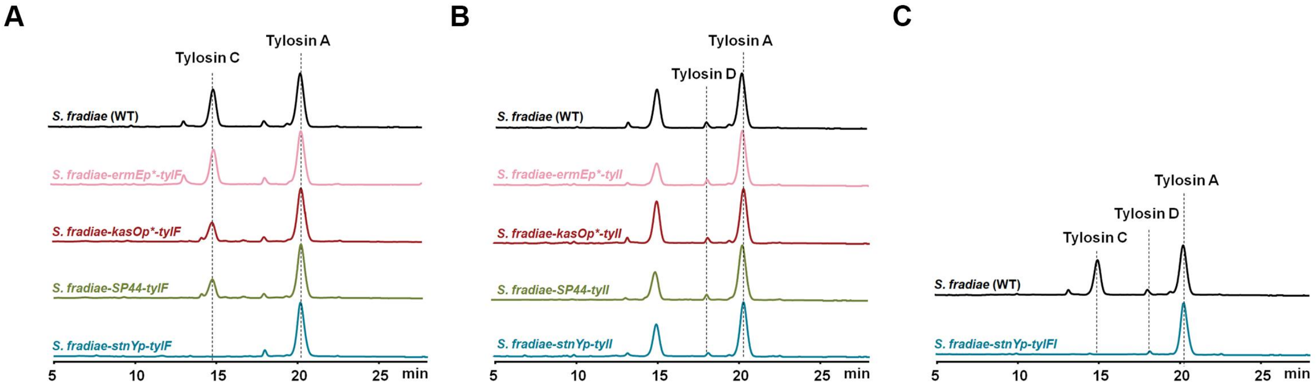

Table S1. Bacterial strains used in this study.

Bacteria strains	Relevant characteristics	Ref/Source
***Streptomyces* strains**		
S. flocculus CGMCC 4.1223	Streptonigrin wild type producing strain	[1]
S. venezuela ISP5230	Wild type, *Streptomyces* host strain for heterologous expression	[2]
S. venezuela-xylE	*S. venezuela* ISP5230 containing pDR3, Apr^{R}	This study
S. venezuela-ermEp-xylE*	*S. venezuela* ISP5230 containing pDR3-*ermEp**, Apr^{R}	This study
S. venezuela-kasOp-xylE*	*S. venezuela* ISP5230 containing pDR3-*kasOp**, Apr^{R}	This study
S. venezuela-SP44-xylE	*S. venezuela* ISP5230 containing pDR3-*SP44*, Apr^{R}	This study
S. venezuela-stnYp-xylE	*S. venezuela* ISP5230 containing pDR3-*stnYp*, Apr^{R}	This study
S. coelicolor M1154	A derivative of the wild-type strain A3(2) lacking plasmids SCP1 and SCP2	[3]
S. coelicolor-xylE	*S. coelicolor* M1154 containing pDR3, Apr^{R}	This study
S. coelicolor-ermEp-xylE*	*S. coelicolor* M1154 containing pDR3-*ermEp**, Apr^{R}	This study
S. coelicolor-kasOp-xylE*	*S. coelicolor* M1154 containing pDR3-*kasOp**, Apr^{R}	This study
S. coelicolor-SP44- xylE	*S. coelicolor* M1154 containing pDR3-*SP44*, Apr^{R}	This study
S. coelicolor-stnYp- xylE	*S. coelicolor* M1154 containing pDR3- *stnYp*, Apr^{R}	This study
S. lividans TK24	*A plasmid-free derivative of S. lividans 66*	[4]
S. lividans-xylE	*S. lividans* TK24 containing pDR3, Apr^{R}	This study
S. lividans-ermEp-xylE*	*S. lividans* TK24 containing pDR3-*ermEp**, Apr^{R}	This study
S. lividans-kasOp-xylE*	*S. lividans* TK24 containing pDR3-*kasOp**, Apr^{R}	This study

S. lividans-SP44-xylE	*S. lividans* TK24 containing pDR3-*SP44*, Apr^R	This study
S. lividans-stnYp-xylE	*S. lividans* TK24 containing pDR3-*stnYp*, Apr^R	This study
S. lividans-pSET152	*S. lividans* TK24 containing pSET152, Apr^R	This study
*S. lividans-ermEp**-anm	*S. lividans* TK24 containing pSET-*ermEp**-anm, Apr^R	This study
*S. lividans-kasOp**-anm	*S. lividans* TK24 containing pSET-*kasOp**-anm, Apr^R	This study
S. lividans-SP44-anm	*S. lividans* TK24 containing pSET-*SP44*-anm, Apr^R	This study
S. lividans-stnYp-anm	*S. lividans* TK24 containing pSET-*stnYp*-anm, Apr^R	This study
S. lividans-pJTU2554	*S. lividans* TK24 containing pJTU2554, Apr^R	This study
*S. lividans-ermEp**-YM	*S. lividans* TK24 containing YM-*ermEp**, Apr^R	This study
*S. lividans-kasOp**-YM	*S. lividans* TK24 containing YM-*kasOp**, Apr^R	This study
S. lividans-SP44-YM	*S. lividans* TK24 containing YM-*SP44*, Apr^R	This study
S. lividans-stnYp-YM	*S. lividans* TK24 containing YM-*stnYp*, Apr^R	This study
S. albidoflavus J1074	A derivative of *S. albidoflavus* G1 defective in the *Sal*IG1 restriction–modification system.	[5]
S. albus-pDR3	*S. albus* J1074 containing pDR3, Apr^R	This study
S. albus-ermEp- xylE*	*S. albus* J1074 containing pDR3-*ermEp**, Apr^R	This study
S. albus-kasOp- xylE*	*S. albus* J1074 containing pDR3-*kasOp**, Apr^R	This study
S. albus-SP44- xylE	*S. albus* J1074 containing pDR3-*SP44*, Apr^R	This study
S. albus-$stnYp_{339}$- *xylE*	*S. albus* J1074 containing pDR3-$stnYp_{339}$, Apr^R	This study
S. albus-$stnYpup_{150}$- *xylE*	*S. albus* J1074 containing pDR3-$stnYp_{150}$, Apr^R	This study
S. albus-$stnYpup_{100}$- *xylE*	*S. albus* J1074 containing pDR3-$stnYp_{100}$, Apr^R	This study
S. albus-$stnYpup_{80}$- *xylE*	*S. albus* J1074 containing pDR3-$stnYp_{80}$, Apr^R	This study

S. albus-stnYpup$_{60}$*- xylE*	*S. albus* J1074 containing pDR3-*stnYp*$_{60}$, Apr^R	This study
S. albus-stnYpup$_{30}$*- xylE*	*S. albus* J1074 containing pDR3-*stnYp*$_{30}$, Apr^R	This study
S. albus-stnYpup$_{10}$*- xylE*	*S. albus* J1074 containing pDR3-*stnYp*$_{10}$, Apr^R	This study
S. albus-indC	*S. albus* J1074 containing pSET-*indC*, Apr^R	This study
S. albus-ermEp-indC*	*S. albus* J1074 containing pSET-*ermEp*-indC*, Apr^R	This study
S. albus-kasOp-indC*	*S. albus* J1074 containing pSET-*kasOp*-indC*, Apr^R	This study
S. albus-SP44-indC	*S. albus* J1074 containing pSET-*SP44-indC*, Apr^R	This study
S. albus-stnYp-indC	*S. albus* J1074 containing pSET-*stnYp-indC*, Apr^R	This study
S. albus-pSET152	*S. albus* J1074 containing pSET152, Apr^R	This study
*S. albus-ermEp**-anm	*S. albus* J1074 containing pSET-*ermEp**-anm, Apr^R	This study
*S. albus-kasOp**-anm	*S. albus* J1074 containing pSET-*kasOp**-anm, Apr^R	This study
S. albus-SP44-anm	*S. albus* J1074 containing pSET-*SP44*-anm, Apr^R	This study
S. albus-stnYp-anm	*S. albus* J1074 containing pSET-*stnYp*-anm, Apr^R	This study
S. albus-pJTU2554	*S. albus* J1074 containing pJTU2554, Apr^R	This study
*S. albus-ermEp**-YM	*S. albus* J1074 containing YM-*ermEp**, Apr^R	This study
*S. albus-kasOp**-YM	*S. albus* J1074 containing YM-*kasOp**, Apr^R	This study
S. albus-SP44-YM	*S. albus* J1074 containing YM-*SP44*, Apr^R	This study
S. albus-stnYp-YM	*S. albus* J1074 containing YM-*stnYp*, Apr^R	This study
Streptomyces fradiae	Industrial strain	
S. fradiae-ermEp-tylF*	*S. fradiae* containing pSET-*ermEp*-tylF*, Apr^R	This study
S. fradiae-kasOp-tylF*	*S. fradiae* J1074 containing pSET-*kasOp*- tylF*, Apr^R	This study
S. fradiae-SP44-tylF	*S. fradiae* J1074 containing pSET-*SP44- tylF*, Apr^R	This study

S. fradiae-stnY-tylF	*S. fradiae* J1074 containing pSET-*stnYp*- *tylF*, Apr^{R}	This study
S. fradiae-ermEp-tylI*	*S. fradiae* containing pSET-*ermEp*-tylI*, Apr^{R}	This study
S. fradiae-kasOp-tylI*	*S. fradiae* J1074 containing pSET-*kasOp**- *tylI*, Apr^{R}	This study
S. fradiae-SP44-tylI	*S. fradiae* J1074 containing pSET-*SP44*- *tylI*, Apr^{R}	This study
S. fradiae-stnY-tylI	*S. fradiae* J1074 containing pSET-*stnYp*- *tylI*, Apr^{R}	This study
S. fradiae-stnY-tylFI	*S. fradiae* J1074 containing pSET-*stnYp*- *tylFI*, Apr^{R}	This study
***E. coli* strains**		
E. coli DH5α	F^{-} φ80*lac*ZΔM15Δ(*lac*ZYA-*arg*F) U169 *rec*A1 *end*A1 *hsd*R17(r$_{K}^{-}$,m$_{K}^{+}$) *pho*A *sup*E44 λ^{-} *thi*-1 *gyr*A96 *rel*A1	ThermoFisher Scientific
E. coli ET12567/pUZ8002	*dam dcm hsdS cat tet*/pUZ8002	[6]
E. coli BW25113/pIJ790	K-12 derivative; Δ*araBAD* Δ*rhaBAD*	[7]

Table S2. Plasmids used in this study.

Plasmid	Relevant characteristics	Ref/source
**Plasmids**		
pDR3	Double-reporter vector containing a promoterless *xylE*-*neo* cassette, Apr^{R}	[8]
pDR3-*ermEp**	A derivative of pDR3 containing the promoter *ermEp**, Apr^{R}	This study
pDR3-*kasOp**	A derivative of pDR3 containing the promoter *kasOp**, Apr^{R}	This study
pDR3-*SP44*	A derivative of pDR3 containing the promoter *SP44*, Apr^{R}	This study
pDR3-$stnYp_{339}$	A derivative of pDR3 containing the promoter $stnYp_{339}$, Apr^{R}	This study
pDR3-$stnYpup_{150}$	A derivative of pDR3 containing the promoter $stnYpup_{150}$, Apr^{R}	This study
pDR3-$stnYpup_{100}$	A derivative of pDR3 containing the promoter $stnYpup_{100}$, Apr^{R}	This study
pDR3-$stnYpup_{80}$	A derivative of pDR3 containing the promoter $stnYpup_{80}$, Apr^{R}	This study
pDR3-$stnYpup_{60}$ (pDR3-*stnYp*)	A derivative of pDR3 containing the promoter $stnYpup_{60}$(*stnYp*), Apr^{R}	This study
pDR3-$stnYpup_{30}$	A derivative of pDR3 containing the promoter $stnYpup_{30}$, Apr^{R}	This study

pDR3-*stnYpup*$_{10}$	A derivative of pDR3 containing the promoter *stnYpup*$_{10}$, Apr^R	This study
pSET152	Integrative vector, Apr^R	[9]
pSET-*ermEp**-*indC*	A derivative of pSET-*indC* containing the promoter *ermEp**, Apr^R	This study
pSET-*kasOp**-*indC*	A derivative of pSET-*indC* containing the promoter *kasOp**, Apr^R	This study
pSET-*SP44*-*indC*	A derivative of pSET-*indC* containing the promoter *SP44*, Apr^R	This study
pSET-*stnYp*-*indC*	A derivative of pSET-*indC* containing the promoter *stnYp*, Apr^R	This study
pSET-anmBCDE	A derivative of pSET152 containing the entire Aureonuclemycin gene cluster, Apr^R	[10]
pSET-*ermEp**-anm	A derivative of pSET-anmBCDE with replacement of the native *anmB* promoter by *ermEp**, Apr^R	This study
pSET-*kasOp**-anm	A derivative of pSET-anmBCDE with replacement of the native *anmB* promoter by *kasOp**, Apr^R	This study
pSET-*SP44*-anm	A derivative of pSET-anmBCDE with replacement of the native *anmB* promoter by *SP44*, Apr^R	This study
pSET-*stnYp*-anm	A derivative of pSET-anmBCDE with replacement of the native *anmB* promoter by *stnYp*, Apr^R	This study
pTG1104	Conjugative and *Φ*C31-integrative cosmid containing the YM-216391 gene cluster, Apr^R	[11]

YM-*ermEp**	A derivative of pTG1104 with replacement of the native *ymI* promoter by *emEp**, Apr^R , Kan^R	This study
YM-*kasOp**	A derivative of pTG1104 with replacement of the native *ymI* promoter by *kasOp**, Apr^R , Kan^R	This study
YM-*SP44*	A derivative of pTG1104 with replacement of the native *ymI* promoter by *SP44*, Apr^R , Kan^R	This study
YM-*stnYp*	A derivative of pTG1104 with replacement of the native *ymI* promoter by *stnYp*, Apr^R , Kan^R	This study
pSET-*ermEp**-*tylF*	A derivative of pSET152 with overexpression of *tylF* by *ermEp**, Apr^R	This study
pSET-*kasOp**-*tylF*	A derivative of pSET152 with overexpression of *tylF* by *kasOp**, Apr^R	This study
pSET-*SP44-tylF*	A derivative of pSET152 with overexpression of *tylF* by *SP44*, Apr^R	This study
pSET-*stnYp-tylF*	A derivative of pSET152 with overexpression of *tylF* by *stnYp*, Apr^R	This study
pSET-*ermEp**-*tylI*	A derivative of pSET152 with overexpression of *tylI* by *ermEp**, Apr^R	This study
pSET-*kasOp**-*tylI*	A derivative of pSET152 with overexpression of *tylI* by *kasOp**, Apr^R	This study
pSET-*SP44-tylI*	A derivative of pSET152 with overexpression of *tylI* by *SP44*, Apr^R	This study
pSET-*stnYp-tylI*	A derivative of pSET152 with overexpression of *tylI* by *stnYp*, Apr^R	This study

pSET-*stnYp*-*tylFI*	A derivative of pSET152 with overexpression of *tylFI* by *stnYp*, Apr^R	This study
pIJ790	λ-RED (*gam, bet, exo*), *cat*, *araC*, *rep101*^{ts}	[12]
pJTU4659	containing the *neo* cassette used for replace the gene using PCR targeting and λ-Red-mediated recombination, Kan^R	This lab
pJTU2554	3.9-kb *XbaI/XhoI* fragment from pSET152 that was ligated with 5.5-kb *XbaI/XhoI* fragment, Apr^R	[13]

82 Apr^R apramycin resistance, Kan^R kanamycin resistance, Amp^R ampicillin resistance.

Table S3. Primers used in this study

Primer	Sequence (5′-3′)[a, b]	Purpose
stnYp$_{399}$F	aggaggaactatatccgcgggatcctgtgcgagcataacctctgc	For construction of plasmids pDR3-*stnYp*$_{339}$, pDR3-*stnYpup*$_{150}$, pDR3-*stnYpup*$_{100}$, pDR3-*stnYpup*$_{80}$, pDR3-*stnYpup*$_{60}$, pDR3-*stnYpup*$_{30}$, pDR3-*stnYpup*$_{10}$, pDR3-*ermEp**, pDR3-*kasOp**, and pDR3-*SP44*.
stnYpup$_{150}$F	aggaggaactatatccgcgggatccccggtggcgggccgaggcg	
stnYpup$_{100}$F	aggaggaactatatccgcgggatccgccgtggcggggcagggag	
stnYpup$_{80}$F	aggaggaactatatccgcgggatccggcgggggacggcatcgtc	
stnYpup$_{60}$F	aggaggaactatatccgcgggatccgcatccggtccgcgaaggat	
stnYpup$_{30}$F	aggaggaactatatccgcgggatccttctccatgaggtcgccgcg	
stnYpup$_{10}$F	aggaggaactatatccgcgggatccgcgggcatgcttggcgtgcg	
stnYpR	tcacctcttcaactcagatactagtctcttcagcagtcatgtgag	
ExylEF	aggaggaactatatccgcgggatccgcgagtgtccgttcgagtgg	
ExylER	tcacctcttcaactcagatactagtcgctggatcctaccaaccgg	
OxylEF	aggaggaactatatccgcgggatcctgttcacattcgaacggtct	
OxylER	tcacctcttcaactcagatactagtaactcccccagtcctgcacg	
PxylEF	aggaggaactatatccgcgggatcctgttcacattcgaacggtct	
PxylER	tcacctcttcaactcagatactagtaactcccccagtcctgcacg	
indCF	gcatacgtactagttaattaattaagccgtacccatcgcagatca	For construction of plasmids pSET- *indC*, pSET-*ermEp*-indC*, pSET-*kasOp*-indC*, pSET-*SP44-indC*, and pSET-*stnYp-indC*.
indCR	cgggctttttctgtgtttccgcgtctaggcgaagaggtccaagg	
EindCF	tagccatggatgcatacgtactagtgcgagtgtccgttcgagtgg	
EindCR	ggtacggcttaattaattaactagtcgctggatcctaccaaccgg	
OindCF	tagccatggatgcatacgtactagttgttcacattcgaacggtct	
OindCR	ggtacggcttaattaattaactagtaactcccccagtcctgcacg	
PindCF	tagccatggatgcatacgtactagttgttcacattcgaacggtct	
PindCR	ggtacggcttaattaattaactagtaactcccccagtcctgcacg	
YindCF	tagccatggatgcatacgtactagtgcatccggtccgcgaaggat	

YindCR	cgggtggcggcgtgctggtgctcatcgttgacgatcccccgagtg	
152EF	gcttgggctgcaggtcgactctagagcgagtgtccgttcgagtgg	For construction of plasmids pSET-*ermEp**-aur, pSET-*kasOp**-aur, pSET-*SP44*-aur, and pSET-*stnYp*-aur.
aurER	gtccgggcacacggaatcgactagtcgctggatcctaccaaccgg	
EaurF	cggttggtaggatccagcgactagtcgattccgtgtgcccggacG	
152aurR	cgatatcgcgcgcggccgcggatcctcatacggcggccatcgcgt	
152OF	gcttgggctgcaggtcgactctagatgttcacattcgaacggtct	
aurOR	gtccgggcacacggaatcgactagtaactcccccagtcctgcacg	
OaurF	gtgcaggactgggggagttactagtcgattccgtgtgcccggacg	
152aurR	cgatatcgcgcgcggccgcggatcctcatacggcggccatcgcgt	
152PF	gcttgggctgcaggtcgactctagatgttcacattcgaacggtct	
aurPR	gtccgggcacacggaatcgactagtaactcccccagtcctgcacg	
PaurF	gtgcaggactgggggagttactagtcgattccgtgtgcccggacg	
152aurR	cgatatcgcgcgcggccgcggatcctcatacggcggccatcgcgt	
152YF	gcttgggctgcaggtcgactctagagcatccggtccgcgaaggat	
aurYR	ggcacacggaatcgactagtctcttcagcagtcatgtgagcatgc	
YaurF	tcacatgactgctgaagagactagtcgattccgtgtgcccggacg	
152aurR	cgatatcgcgcgcggccgcggatcctcatacggcggccatcgcgt	
ymkanF	tccccgccgcaccgtacggcaggctattccggggatccgtcgacc	For construction of plasmids YM-*ermEp**, YM-*kasOp**, YM-*SP44*, and YM-*stnYp.*
EkanR	agccgccactcgaacggacactcgctgtaggctggagctgcttcg	
kanEF	aacttcgaagcagctccagcctacagcgagtgtccgttcgagtgg	
ymER	acagcttctccggtatctgctaaggcgctggatcctaccaaccgg	
ymkanF	tccccgccgcaccgtacggcaggctattccggggatccgtcgacc	
OkanR	agcagagaccgttcgaatgtgaacatgtaggctggagctgcttcg	
kanOF	aacttcgaagcagctccagcctacatgttcacattcgaacggtct	
ymOR	acagcttctccggtatctgctaaggaactcccccagtcctgcacg	
ymkanF	tccccgccgcaccgtacggcaggctattccggggatccgtcgacc	
PkanR-	agcagagaccgttcgaatgtgaacatgtaggctggagctgcttcg	
kanPF	aacttcgaagcagctccagcctacatgttcacattcgaacggtct	
ymPR	acagcttctccggtatctgctaaggaactcccccagtcctgcacg	

ymkanF	tccccgccgcaccgtacggcaggctattccggggatccgtcgacc	
YkanR	cggccatccttcgcggaccggatgctgtaggctggagctgcttcg	
kanY-F	aacttcgaagcagctccagcctacagcatccggtccgcgaaggat	
ymYR	acagcttctccggtatctgctaaggctcttcagcagtcatgtgag	
EtylFF	gtaggatccaccacctctcccgaccccgcg	For construction of plasmids pSET-*ermEp**-*tylF*, pSET-*kasOp**-*tylF*, pSET-*SP44*-*tylF*, pSET-*stnYp*-*tylF*, pSET-*ermEp**-*tylI*, pSET-*kasOp**-*tylI*, pSET-*SP44*-*tylI*, pSET-*stnYp*-*tylI*, and pSET-*stnYp*-*tylFI*.
152tylFR	cagctatgacatgattacactagtgaattctcagccgctgtgccgcca	
152EF	aggaggaactatatccgcgggatccgtcgactctagtatgcatgc	
tylFER	ggagaggtggtggatcctaccaaccggcacg	
OtylFF	actgggggagttccacctctcccgaccccgcg	
152tylFR	cagctatgacatgattacactagtgaattctcagccgctgtgccgcca	
152OF	aggaggaactatatccgcgggatcctgttcacattcgaacggtctc	
tylFOR	agaggtggaactcccccagtcctgcacgc	
PtylFF	tggtgtaccacctctcccgaccccgcgag	
152tylFR	cagctatgacatgattacactagtgaattctcagccgctgtgccgcca	
152PF	aggaggaactatatccgcgggatcctgttcacattcgaacggtctc	
tylFPR	tcgggagaggtggtacaccagactttacaacaccgc	
YtylFF	tgctgaagagccacctctcccgaccccgcg	
152tylFR	cagctatgacatgattacactagtgaattctcagccgctgtgccgcca	
152YF	aggaggaactatatccgcgggatccgcatccggtccgcgaaggat	
tylFYR	ggagaggtggctcttcagcagtcatgtgagcatgctag	
RBStylIF	atcgacccgctactggaggacccatgacgacacagacgctcgaag	
152tylIR	acatgattacactagtgaattctcagtggcgggtgacggtgaccgg	
tylI152F	gaattcactagtgtaatcatgtcatagctg	
tylIRBSR	tcctccagtagcgggtcgatcg	
tylItylFF	tcaccgtcacccgccactgacatatgccacctctcccgaccccgcg	
152tylFR	ggaaacagctatgacatgattacactagtgaattctcagccgctgtg	
EtylFF	gtaggatccaccacctctcccgaccccgcg	
152tylFR	cagctatgacatgattacactagtgaattctcagccgctgtgccgcca	
152EF	aggaggaactatatccgcgggatccgtcgactctagtatgcatgc	
tylFER	ggagaggtggtggatcctaccaaccggcacg	

OtylFF	actgggggagttccacctctcccgaccccgcg	
152tylFR	cagctatgacatgattacactagtgaattctcagccgctgtgccgcca	
152OF	aggaggaactatatccgcgggatcctgttcacattcgaacggtctc	
tylFOR	agaggtggaactcccccagtcctgcacgc	
PtylFF	tggtgtaccacctctcccgaccccgcgag	
152tylFR	cagctatgacatgattacactagtgaattctcagccgctgtgccgcca	
152PF	aggaggaactatatccgcgggatcctgttcacattcgaacggtctc	
tylFPR	tcgggagaggtggtacaccagactttacaacaccgc	
YtylFF	tgctgaagagccacctctcccgaccccgcg	
152tylFR	cagctatgacatgattacactagtgaattctcagccgctgtgccgcca	
152YF	aggaggaactatatccgcgggatccgcatccggtccgcgaaggat	
tylFYR	ggagaggtggctcttcagcagtcatgtgagcatgctag	
StnYF	gtgaggcagttcaacgtctac	5’ RACE verification of transcription start sites for promoter *stnY.*
StnYR	tcagctcttgagcggcgcct	
StnR1 (463)	gaccatccgctgggtgccgtag	
StnR2 (196)	gccttcgacaccctgggtcgtca	
StnR3 (145)	tgtctcgttcttgccgctggtgc	
StnR4 (89)	agcgccgacagcgacagttcg	
ymIAF	ccggcagggaggcgccttct	RT-PCR co-transcription analyses of genes in the *ym* cluster
ymIAR	gcgacgtccaggtcgtcctcttcc	
ymADF	tgaccgctgagatcgaggaagtcg	
ymADR	tgtacctgatggcgtagtag	
ymDEF	gcggggcgatgcggtcaccg	
ymDER	cgtggcgtcgtgatccgtgaggac	
ymEB1F	ccgcggcctgacgagttcctcc	
ymEB1R	tttcggtcgttccgccgggg	
ymB1C1F	gttcacctcgccgctgtccttgg	
ymB1C1R	ggaggcgtccgccggcagcg	
ymC1FF	cgcggtgctgttcgcgttcg	
ymC1FR	cgagaccggcccgccccgcc	

ymFBCF	gccgcgatcgtacggagtcttgg	
ymFBCR	ggaccgaggtacgccgtcca	
xylEF	caggtgctggacgaaaatgg	qRT-PCR analyses of gene *xylE*, *ymA*, *ymE*, *ymC1*, *ymBC*, and *hrdB*.
xylER	gtcatggagatcaggtcggc	
hrdBF	cgggatctggtcagcttcg	
hrdBR	ccgagtcccagtctgtgatg	
ymAF	cggagaacatcatgaccgct	
ymAR	tccaggtcgtcctcttccag	
ymEF	cccgaaatccccgacattca	
ymER	atgcactccatcaccgtctg	
ymC1F	gaggcgtggttctacgagat	
ymC1R	catcgtccaggagtacgtcg	
ymBCF	gtatctggagcgcgggtatc	
ymBCR	agcagatacagagcctggga	

References

1. Xu F, Kong D, He X, Zhang Z, Han M, Xie X, et al. Characterization of streptonigrin biosynthesis reveals a cryptic carboxyl methylation and an unusual oxidative cleavage of a N-C bond. J Am Chem Soc. 2013;135(5):1739-48.

2. Yang K HL, Vining LC. Regulation of jadomycin B production in *Streptomyces venezuelae* ISP5230: involvement of a repressor gene, jadR2. J Bacteriol. 1995;177(21):6111-7.

3. Gomez-Escribano JP, Bibb MJ. Engineering *Streptomyces coelicolor* for heterologous expression of secondary metabolite gene clusters. Microb Biotechnol. 2011;4(2):207-15.

4. Hopwood DA, Kieser T, Wright HM, Bibb MJ. Plasmids, recombination and chromosome mapping in *Streptomyces lividans* 66. J Gen Microbiol. 1983;129(7):2257-69.

5. Chater KF, Wilde LC. *Streptomyces albus* G mutants defective in the SalGI restriction-modification system. J Gen Microbiol. 1980;116(2):323-34.

6. Paget MS, Chamberlin L, Atrih A, Foster SJ, Buttner MJ. Evidence that the extracytoplasmic function sigma factor sigmaE is required for normal cell wall structure in *Streptomyces coelicolor* A3(2). J Bacteriol. 1999;181(1):204-11.

7. Datsenko KA, Wanner BL. One-step inactivation of chromosomal genes in *Escherichia coli* K-12 using PCR products. Proc Natl Acad Sci. 2000;97(12):6640-5.

8. Wang W, Li X, Wang J, Xiang S, Feng X, Yang K. An engineered strong promoter for *streptomycetes*. Appl Enaviron Microbiol. 2013;79(14):4484-92.

9. Bierman M, Logan R, Brien K, Seno ET, Rao RN, Schoner BE. Plasmid cloning vectors for the conjugal transfer of DNA from *Escherichia coli* to *Streptomyces* spp. Gene. 1992;116:43-9.

10. Pan HX, Chen Z, Zeng T, Jin WB, Geng Y, Lin GM, et al. Elucidation of the Herbicidin Tailoring Pathway

Offers Insights into Its Structural Diversity. Org Lett. 2019;21(5):1374-8.

11. Jian XH, Pan HX, Ning TT, Shi YY, Chen YS, Li Y, et al. Analysis of YM-216391 biosynthetic gene cluster and improvement of the cyclopeptide production in a heterologous host. ACS Chem Biol. 2012;7:646-51.

12. Gust B, Challis GL, Fowler K, Kieser T, Chater KF. PCR-targeted *Streptomyces* gene replacement identifies a protein domain needed for biosynthesis of the sesquiterpene soil odor geosmin. Proc Natl Acad Sci. 2003;100(4):1541-6.

13. Li L, Xu Z, Xu X, Wu J, Zhang Y, He X, et al. The mildiomycin biosynthesis: initial steps for sequential generation of 5-hydroxymethylcytidine 5'-monophosphate and 5-hydroxymethylcytosine in *Streptoverticillium rimofaciens* ZJU5119. Chembiochem. 2008;9:1286-94.
